# ICAMs are dispensable for influenza clearance and anti-viral humoral and cellular immunity

**DOI:** 10.3389/fimmu.2022.1041552

**Published:** 2023-02-21

**Authors:** Stav Kozlovski, Ofer Regev, Anita Sapoznikov, Marina Kizner, Hagit Achdout, Ekaterina Petrovich-Kopitman, Jacob Elkahal, Yoseph Addadi, Fernanda Vargas E. Silva Castanheira, Sara W. Feigelson, Paul Kubes, Noam Erez, Natalio Garbi, Ronen Alon

**Affiliations:** ^1^ Department of Immunology and Regenerative Biology, Weizmann Institute of Science, Rehovot, Israel; ^2^ Department of Biochemistry and Molecular Genetics, Israel Institute for Biological Research, Ness-Ziona, Israel; ^3^ Life Sciences Core Facilities, Weizmann Institute of Science, Rehovot, Israel; ^4^ Department of Molecular Cell Biology, Weizmann Institute of Science, Rehovot, Israel; ^5^ Department of Pharmacology and Physiology, Cumming School of Medicine, University of Calgary, Calgary, AB, Canada; ^6^ Department of Cellular Immunology, Institute of Experimental Immunology Medical Faculty, University of Bonn, Bonn, Germany

**Keywords:** leukocyte trafficking, integrins, endothelium, inflammation, memory

## Abstract

αLβ2 (LFA-1) mediated interactions with ICAM-1 and ICAM-2 predominate leukocyte-vascular interactions, but their functions in extravascular cell-cell communications is still debated. The roles of these two ligands in leukocyte trafficking, lymphocyte differentiation, and immunity to influenza infections were dissected in the present study. Surprisingly, double ICAM-1 and ICAM-2 knock out mice (herein ICAM-1/2^-/-^ mice) infected with a lab adapted H1N1 influenza A virus fully recovered from infection, elicited potent humoral immunity, and generated normal long lasting anti-viral CD8^+^ T cell memory. Furthermore, lung capillary ICAMs were dispensable for both NK and neutrophil entry to virus infected lungs. Mediastinal lymph nodes (MedLNs) of ICAM-1/2^-/-^ mice poorly recruited naïve T cells and B lymphocytes but elicited normal humoral immunity critical for viral clearance and effective CD8^+^ differentiation into IFN-γ producing T cells. Furthermore, whereas reduced numbers of virus specific effector CD8^+^ T cells accumulated inside infected ICAM-1/2^-/-^ lungs, normal virus-specific T_RM_ CD8^+^ cells were generated inside these lungs and fully protected ICAM-1/2^-/-^ mice from secondary heterosubtypic infections. B lymphocyte entry to the MedLNs and differentiation into extrafollicular plasmablasts, producing high affinity anti-influenza IgG2a antibodies, were also ICAM-1 and ICAM-2 independent. A potent antiviral humoral response was associated with accumulation of hyper-stimulated cDC2s in ICAM null MedLNs and higher numbers of virus-specific T follicular helper (Tfh) cells generated following lung infection. Mice selectively depleted of cDC ICAM-1 expression supported, however, normal CTL and Tfh differentiation following influenza infection, ruling out essential co-stimulatory functions of DC ICAM-1 in CD8^+^ and CD4^+^ T cell differentiation. Collectively our findings suggest that lung ICAMs are dispensable for innate leukocyte trafficking to influenza infected lungs, for the generation of peri-epithelial T_RM_ CD8^+^ cells, and long term anti-viral cellular immunity. In lung draining LNs, although ICAMs promote lymphocyte homing, these key integrin ligands are not required for influenza-specific humoral immunity or generation of IFN-γ effector CD8^+^ T cells. In conclusion, our findings suggest unexpected compensatory mechanisms that orchestrate protective anti-influenza immunity in the absence of vascular and extravascular ICAMs.

## Introduction

The respiratory tract is comprised of distinct epithelial layers and vascular beds including the alveolar gas-exchange surfaces, which contain the largest vascular bed in the body (1). The bronchial and alveolar epithelial cells are key targets for viral infections (2). Infected respiratory epithelial cells triggers multiple sequential immune responses critical for both virus clearance and for the establishment of anti-viral immune memory (3). It is commonly accepted that leukocyte adherence and emigration across pulmonary capillaries is molecularly distinct from the classical multistep cascade of leukocyte adhesion to and emigration across inflamed postcapillary venules (4, 5). When infected with highly pathogenic pneumotropic influenza strains, different innate and adaptive immune cells must be selectively recruited to various compartments of virus-infected upper and lower airways and lung alveoli using distinct trafficking cues. In addition, different virus-specific naïve T and B lymphocytes must enter the main lung draining lymph nodes in which the presentation of virus antigens is executed by professional antigen presenting dendritic cells (6). Comprehensive knowledge of the trafficking signals and specifically the cell adhesion molecules functioning on these distinct vascular beds during influenza infections is still missing. Three of such adhesion molecules ICAM-1 (CD54), ICAM-2 (CD102) and VCAM-1 (CD106) are single chain Ig superfamily transmembrane glycoproteins (1, 7). ICAM-1 and ICAM-2 are ligands of the key leukocyte β2-integrin LFA-1 (8), whereas VCAM-1 is a major vascular ligand for the integrin α4β1 (VLA-4). ICAM-1 and VCAM-1 expression is generally upregulated by inflammatory cytokines, while ICAM-2 (CD102) is constitutively expressed (9, 10).

These three ligands are also constitutively expressed on high endothelial venules (HEVs), specialized postcapillary venules that are the main portal of lymphocyte entry to all lymph nodes (LNs) (11). ICAM-1 and ICAM-2 are also expressed on numerous immune cells, in particular antigen presenting cells (APCs) like DCs, B cells and macrophages (12), and are thought to stabilize functional immune synapses between these cells and other immune cells (13). Nevertheless, the roles of these β2-integrin ligands in the complex communications between distinct lung APCs and T cells necessary for the establishment of early and long-lasting antiviral cellular and humoral immunity have never been investigated in the context of influenza infections.

Multiple studies have suggested the usage of lung ICAMs or their main CD18 (β2) integrin receptors in leukocyte recruitment to the lungs in distinct infections (4). To systematically dissect the roles of the two ICAMs in innate and adaptive immunity to influenza infections, we followed the course of H1N1 influenza infection in WT and in ICAM-1/2^-/-^ mice. Additionally, we addressed the role of α4 integrin interactions in leukocyte entry to distinct lung compartments and lung draining lymph nodes. Strikingly, ICAM-1/2^-/-^ mice normally recovered from infection, elicited potent humoral immunity, and generated long lasting cellular immunity to both homosubtypic and heterosubtypic variants of influenza A strains. Intravital staining of lung vessels following influenza infection suggested a unique compartmentalization of VCAM-1 and ICAM-1, respectively, between virus infected peribronchial vessels and lung capillaries. Nevertheless, vascular ICAMs were not obligatory for neutrophil or NK cell recruitment to virus infected lungs. On the other hand, vascular ICAMs promoted T and B lymphocyte entry to lung draining lymph nodes both prior to and after infection. Strikingly, however, viral Ag dependent proliferation and differentiation of naïve CD4^+^ T cells into effector Tfh cells took place normally and coincided with the appearance of hyperactive cDC2 subsets which accumulated in ICAM-1/2^-/-^ MedLNs of influenza-infected mice. Furthermore, using a newly developed DC specific ICAM-1 KO mouse, we found normal early Ag specific naïve CD4^+^ and CD8^+^ T cell priming by influenza expressed antigens, normal Ag stimulated CD8^+^ T cell binding to ICAM-1 deficient DCs and intact CD8^+^ lymphocyte differentiation. Our work suggests unexpected compensatory mechanisms facilitated by vascular and DC ligands other than ICAMs which recruit innate and adaptive leukocytes to infected lungs, and elicit protective anti-influenza adaptive immunity.

## Materials and methods

### Mice

Mice (all on C57BL/6 background) were maintained in a pathogen-free facility at the Weizamnn Institute of Science (WIS) animal facility and all animal procedures were approved by the WIS Institutional Animal Care and Use Committee (application numbers: 14350519-3, 07491118-1, 02640418-3, 35830617-2 and 24610116-2). 8-to-14-week-old male and female mice were used in all the experiments. The ICAM-1/2^-/-^ mice were a kind gift of Prof. Britta Engelhardt and were back crossed for 10 generations. The CD45.1 OT-I and OT-II TCR transgenic mice harboring OVA-specific CD8^+^ and CD4^+^ T cells, respectively, were kindly provided by Prof. Steffen Jung (WIS). The Conditional ICAM-1 Knockout mice (ICAM-1fl/fl) were generated in our lab using CRISPR-Cas9. These mice were crossed with CD11c-Cre mice (14) provided by Prof. Steffen Jung to generate the CD11C-Cre : ICAM-1^fl/fl^ mice (DC^ΔICAM-1^ mice). Mice were anesthetized prior to all infections/immunizations by I.P. ketamine/xylazine (10 mg/kg) injections. At the endpoint of each experiment, prior to sample collections and organ harvesting, mice were euthanized by I.P. sodium pentobarbital (200 mg/kg) injections.

### Organ preparation for flow cytometry

Bone marrow was extracted from the femurs and tibias of mice as described (15). Spleens were harvested, placed on a 40 μm cell strainer in a 100x15 mm petri dish with 5 mL 1X PBS and crushed with a plunger to dissociate it into the dish. The suspension was pipetted into a 15 mL tube and centrifuged at 394 x g for 5 minutes at 4°C. RBCs were subsequently lysed with an RBC lysis buffer (Sigma Aldrich, Cat. R7757) and the cells re-suspended in ice-cold flow cytometry buffer (PBS, 1% BSA, 0.1% Na azide and 5 mM EDTA). Blood samples were collected from the retro-orbital sinus of the mice. Single cell suspensions were prepared using the BD Pharm Lyse (BD Bioscience, Cat. 555899) lysing buffer according to the manufacturer’s instruction (BD Bioscience). Samples were centrifuged at 394 x g for 5 minutes at 4°C the blood samples were re-suspended in ice-cold flow cytometry buffer.

### Isolation of BAL fluid and preparation of total lung cell suspension for flow cytometry

Mice were euthanized, lungs were transcardially perfused (10 mL 1X PBS) and BAL fluid (BALF) was collected twice with 5 mM EDTA/PBS aliquots introduced through a tracheal catheter. The BAL fluid was centrifuged at 394 x g for 5 minutes at 4°C. For preparation of lung cell suspensions, lungs were extracted, minced and incubated in RPMI-1640 containing collagenase type 4 (1.5 mg/mL) and DNase I (20 μg/mL) at 37°C for 45 minutes. The lung cell suspensions were transferred through a 100 μm cell strainer and centrifuged at 394 x g for 5 minutes at 4°C. RBCs were subsequently lysed with an RBC lysis buffer (Sigma Aldrich, Cat. R7757). The cells from both BALF and lungs were re-suspended in ice-cold buffer and analyzed by flow cytometry. For analysis of protein surface expression, cells were labeled with fluorescent conjugated antibodies (10 μg/mL), incubated on ice for 20-30 minutes, centrifuged at 394 x g for 5 minutes at 4°C and resuspended in ice-cold flow cytometry buffer (PBS, 1% BSA, 0.1% Na azide and 5 mM EDTA). Cells were analyzed on either an LSR-II Flow Cytometer (BD Biosciences) or a CytoFLEX flow cytometer (Beckman Coulter). Intracellular staining for BCL6 and Foxp3 was performed using the True-Nuclear™ Transcription Factor Buffer Set (Biolegend, 424401) according to the manufacturer’s recommendations. Data were analyzed using FlowJo software (BD Biosciences).

### *In vivo* determination of leukocyte recruitment within distinct lung compartments by differential anti-CD45 mAb staining

Mice were administered I.V. with the anti-CD45 mAb clone 30-F11. Mice were euthanized 5 minutes later, lungs were transcardially perfused (10 mL 1X PBS), extracted, and each lung was placed in RPMI-1640 (2.5 mL) containing: 1.5 mg/mL collagenase type 4 (Worthington, LS004188), 20 μg/mL DNase I (Roche, 1010415900) and 1.25 μg of non-labeled anti-CD45 mAb clone 30-F11. Lungs were then minced and incubated at 37°C for 45 minutes.

### Pulmonary intravital microscopy

Imaging and analysis of lung neutrophils was performed as previously described (16). For neutrophil phenotypic quantification the following was defined (17): tethering was defined as a discreet neutrophil interaction with the vascular wall, which arrests its circulatory movement for less than 30 seconds. Crawling was defined as continuous interaction of a neutrophil with the vascular wall for more than 30 seconds, which involves a polarized cell that does not remain stationary. Adhesion was defined as a stationary neutrophil that was not mobile and remained static for at least 30 seconds.

### Light sheet microscopy of lungs

Naïve or 4 days following PR8 infection (I.N. or I.T.) mice were intravitally stained for VCAM-1^+^ and ICAM-1^+^ lung vessels by I.V. injection of 5 μg of Alexa-647 anti-VCAM-1 (CD106) mAb (BD Bioscience, Cat. 561612, clone 429 (MVCAM.A)) or Alexa-568 conjugated anti-ICAM-1(CD54) mAb (Biolegend, Cat. 116101, clone YN1/1.7.4) 5 minutes prior to euthanasia. Mice were transcardially perfused with PBS and the lungs were inflated *via* the trachea with low-gelling agarose (Sigma Aldrich, Cat. A9045), fixed with paraformaldehyde (4% in PBS) for 2 hours, dehydrated, and cleared using ethyl cinnamate (Sigma Aldrich, Cat. 112372) as described (18). Cleared lung lobes were imaged in an Ultramicroscope II (LaVision BioTec) operated by the ImspectorPro software (LaVision BioTec, Bielefeld, Germany) as described (18). For the visualization of PR8 mice were I.T. infected with 3x10^3^ PFU of PR8-mCherry and lungs were imaged 4 days later. The three-dimensional rendering of LSM was performed *via* Imaris software (Oxford Instruments, Abingdon, UK).

### Pathogens

The PR8 strains used in this study were kindly provided by Prof. Natalio Garbi (University Hospital Bonn). The X31 strain was kindly provided by the Shulman lab (WIS). Pseudomonas aeruginosa (strain PAO1) was grown on agar plates overnight at 37°C. Multiple single colonies were picked from plates washed 3 times in sterile PBS and quantified by OD600.

### *In vivo* influenza infections

Anesthetized mice were held in supine and the tongue was gently pulled out using forceps to gain visualization of the larynx. Various PR8 influenza doses (sublethal: 15-155 PFU) and lethal: 3x10^3^ PFU) were delivered. For I.T. administration: PR8 doses were suspended in a total volume of 50 μl of sterile 1X PBS and placed on the base of the tongue while the nose was closed, thus forcing the mice to breathe through the mouth. The nose and tongue were released after at least two breaths had been completed. For I.N. delivery: anesthetized mice were placed in the dorsal recumbency. A pipet tip containing 30 μL/mouse of sublethal doses of PR8, PR8-SIINFEKL or PR8-OVAII (OVA_323–339_ peptide) influenza inoculum in sterile PBS was placed above the mouse nostril and the solution was slowly injected. The mice were validated to have inhaled the entire inoculum drop. For recall infections: infected mice were challenged 40 days after the primary infection. Weight loss was tracked throughout the course of infection.

### Quantification of pulmonary PR8 viral load by real-time quantitative PCR

WT and ICAM-1/2^-/-^ mice were euthanized on day 4 or 12 post I.N. infection with 30 PFU of PR8 influenza. Lungs were snap-frozen and kept at -70°C. Lungs were slowly thawed on ice. Viral genomic PR8-RNA was extracted from the infected lungs and transcribed into cDNA by RT-PCR using NA specific PR8 influenza virus primers (NA Fw: TTAATGAGCTGCCCTGTCGG, NA Rv: CACTTGCTGACCAAGCAACC). The cDNA was used as a template for the RT-qPCR reaction and results are represented as mean PFU equivalent per microgram RNA (mean PFUe/μg RNA). PR8-cDNA samples containing virus-complementary sequences served as quantification standards for the RT-qPCR reaction.

### MedLN preparation for flow cytometry

Isolated LNs were placed on a 40 μm cell strainer, crushed with a plunger and dissociated into a 10x60 mm petri dish. The suspension was transferred into a 96 v-bottom plate (approximately 200ul/well) and the plates were centrifuged at 394 x g for 4 minutes at 4°C. Pellets were collected with two rounds of washes with 100 μl of 1X PBS. Plates were then centrifuged at 394 x g for 5 minutes at 4°C and the cells were re-suspended in ice-cold flow cytometry buffer (PBS, 1% BSA, 0.1% Na azide and 5 mM EDTA). For DC isolation, each LN was placed in a well of a 12-well plate and suspended in 0.5 mL of PBS^+/+^ supplemented with 1 mg/mL Collagenase D (Roche, COLLD-RO). The treated LN was torn with two syringes, incubated for 30 minutes at 37°C and subsequently placed on a 40 μm cell strainer in a 10x60 mm petri dish in the presence of 2 mL PBS.

### Blocking of α4 integrin on innate leukocytes and T cells

Innate leukocyte blocking: On day 0, 60 μgs of the anti-α4 antibody (BioXcell clone: PS/2) or isotype control (BioXcell clone: 2A3) were administered I.V. in a total volume of 150 μl (in 1X PBS) *via* the retro-orbital sinus. 2 hours later, mice were I.N. infected with 30 PFU of the PR8 influenza virus. For the next three days (day 1, 2 and 3), mice were I.V. administered with 30 μg of the same blocking regimen (PS/2 or 2A3). On day 4 mice were euthanized and BALFs of WT and ICAM-1/2^-/-^ mice were analyzed by flow cytometry for neutrophils and NK cells. For T cell blocking: Spleen-derived 10^7^ WT CD45.1 OT-I T cells (2x10^6^ naïve T cells) were incubated for 20 minutes with 30 μg of PS/2 or 2A3 in 100 μl and subsequently administered I.V. *via* the retro-orbital sinus. After 4 hours, an additional 30 μg dose of PS/2 or 2A3 was administered I.V. and 14 hours later the accumulation of the I.V. injected CD45.1 OT-I CD8^+^ T cells in the MedLNs was determined by flow cytometry. In an additional experiment, at this time point ICAM-1/2^-/-^ mice were I.N. administered either with a sublethal dose of PR8-SIINFEKL or PBS and 4 hours later were administered with 30 μg of PS/2 or 2A3 I.V. One or four days later, ICAM-1/2^-/-^ were euthanized and the total number of CD45.1 OT-I CD8^+^ T cells recovered from the MedLNs of the differently treated mice groups was determined by flow cytometry.

### MedLN immunohistochemistry

Naïve WT and ICAM-1/2^-/-^ MedLNs were harvested, fixed for 2 hours at RT in 1% PFA/PBS solution and then placed in 30% sucrose solution O.N. at 4°C. The next day, samples were transferred to a fresh solution of 30% sucrose for an additional 24 hours at 4°C. On the third day, each MedLN was placed in a small cryomold (Fisher scientific, Cat. NC9511236), embedded in OCT compound (Sakura, Cat. 4583), snap frozen on dry ice nuggets and stored at -80°C. Tissue sections (10μm thick) were cut using a cryostat and slides were placed in chilled 70% alcohol for 7 minutes. Prior to staining slides were rehydrated twice with PBS, treated for 5 minutes in 1% SDS/PBS, washed 3 times and blocked with 3% BSA in PBS-T (PBS/0.05% Tween-20). Sections were incubated with primary antibodies diluted in 1% BSA in PBS-T O.N. at 4°C. The following antibodies were used for staining: mouse monoclonal anti-VCAM-1 IgG (clone 429, BD Bioscience, Cat. 550547) and mouse monoclonal anti-PNAd IgM (MECA-79, Biolegend, Cat. 120802). After washing 3 times for 5 minutes in PBS-T at room temperature, the sections were incubated at 4°C O.N. with the following secondary antibodies: anti-rat IgG conjugated to Dylight 549 (Jackson, Cat. 712-505-153) and anti-rat IgM conjugated to Alexa Fluor 647 (Jackson, Cat. 716-605-020). The next day slides were washed and stained with DAPI (1:3000) (Biolegend, Cat. 422801).

### OT-I and OT-II homing to MedLNs at steady state

Spleen derived 2x10^6^ OT-I or 0.1x10^6^ OT-II T cells were labeled with CFSE (5 mM) (Molecular Probes, Cat. 11524217) as described (19) or stained with anti-CD45.1 mAb (Biolegend, Cat. 110708) and subsequently injected I.V. into WT or ICAM-1/2^-/-^ CD45.2 mice. After 18 hours, mice were euthanized, MedLNs were isolated and the numbers of accumulated OT-I CD8^+^ and OT-II CD4^+^ T cells were assessed by flow cytometry.

### OT-II CD4^+^ Tfh effectors and GC B cell generation in MedLNs following PR8-OVAII influenza infection

Spleen-derived 0.1x10^6^ CD45.1 OT-II T cells were injected I.V. into WT, ICAM-1/2^-/-^, DC^ΔICAM-1^ or WT (ICAM-1^fl/fl^) mice. After 18 hours, mice were I.N. infected with a sublethal dose of PR8-OVAII influenza. Mice were euthanized on days 4 or 12 and the totals of the CD45.1 OT-II CD4^+^ Tfh cells were determined by flow cytometry in the MedLNs. The number of GC B cells was determined on day 12 post infection.

### Intra-cytoplasmic cytokine staining of IL-6

Following the isolation of MedLNs, single cell suspensions were resuspended with 100ug/mL of LPS (Sigma Aldrich Cat. L2630-100MG) in BLAST medium (RPMI-1640 medium with 10% FCS, b-mercaptoethanol (50uM), L-glutamine, Sodium Pyruvate (2 mM) and PSA (2 mM)) and incubated for 3 hours at 37°C. All cells were treated for 1.5 hours at 37°C with Brefeldin A solution (1:1,000, Biolegend, Cat. 420601) and Monensin (1:1000, Biolegend, Cat. 420701). Cells were labeled with the following mAb mixture anti-CD45 mAb (Biolegend, Cat. 103108, clone 30-F11), anti-CD11c mAb (Biolegend, Cat. 117322, clone), anti-CD103 mAb (Biolegend, Cat. 121433, clone 2E7), anti-CD11b mAb (Biolegend, Cat. 101228, clone M1/70), anti-CD8 mAb (Biolegend, Cat. 100712, clone 53-6.7), fixed, permeabilized, and subjected to intracellular IL-6 staining with the anti-IL-6 mAb (Biolegend, Cat. 504504, clone MP5-20F3) using the Cytofix/Cytoperm kit (BD Biosciences, Cat. BD 554714).

### ELISA detection of serum IgG and IgG2a antibodies against PR8 influenza virus

WT and ICAM-1/2^-/-^ mice were infected with PR8 influenza and sera samples were collected on days 12, 20 or 40 post infection. The levels of PR8 influenza virus-specific serum IgGs were determined by enzyme-linked immunosorbent assay (ELISA). PR8 virus was purified on sucrose gradient (22-50%). Nunc MaxiSorp plates (Thermo Fisher Scientific, USA) were coated with the purified PR8 virus (5x10^5^ PFU/well) in Na_2_CO_3_/NaHCO_3_ buffer (Sigma Aldrich, Cat. C3041) at 4°C overnight. Plates were blocked with ELISA buffer (50 mM Tris, pH 7.6 + 140 mM NaCl + 0.05% Tween 20 + 2% BSA) for 60 minutes at 37°C. Following blocking and washing with PBST buffer (PBS + 0.05% Tween 20), the plates were incubated with serum samples diluted 1:100 to 1:3x10^4^ in ELISA buffer for 1 hour at 37 °C. Following washing, alkaline phosphatase-conjugated anti-mouse IgG (Sigma Aldrich cat. A5153) was used (diluted 1:1000) as a secondary antibody. After washing, P-nitrophenyl phosphate substrate (Sigma, Israel, cat. N2770) was added, and after 60 minutes of substrate incubation at room temperature, the optical density was measured by using a spectrophotometer (SpectraMax 190 microplate reader, Molecular Devices, Sunnyvale, CA), at 405 nm. Each data point represents the mean OD values from the ELISA analysis of three replicates.

### OT-I CD8^+^ T effectors generated in MedLNs and lungs following PR8-SIINFEKL influenza infection

Spleen-derived 2x10^6^ CD45.1 OT-I T cells were labeled with CFSE (5 mM) (Molecular Probes, Cat. 11524217) as described (19) or alternatively identified by anti-CD45.1 staining. The stained cells were injected I.V. into WT, ICAM-1/2^-/-^, DC^ΔICAM-1^ or WT (ICAM-1^fl/fl^) mice. Following 18 hours, mice were I.N. infected with a sublethal dose of the PR8-SIINFEKL influenza virus. Mice were euthanized on days 4 or 7 and the totals of the OT-I CD8^+^ T cells were determined by flow cytometry either in the MedLNs or the lungs of infected mice.

### *In vivo* T cell proliferation and intra-cytoplasmic IFN-γ staining

CFSE labeled OT-I CD8^+^ T cells were recovered from the MedLNs of WT and ICAM-1/2^-/-^ mice 4 or 7 days after sublethal PR8-SIINFEKL infection and lymphocyte proliferation was assessed by CFSE dilution. “Percent Divided” refers to the percentage of the original T cell population that underwent at least one division. “Division Index” is defined as the average number of cell divisions per cell in the original population. For intracellular staining of IFN-γ, cells were stimulated with 50 ng/mL PMA (Sigma Aldrich, Cat. 16561-29-8) and 500 ng/mL Ionomycin (Sigma Aldrich, Cat. 13909) for 1.5 hours at 37 °C and then treated with Brefeldin A (Biolegend, Cat. 420601) and Monensin (Biolegend, Cat. 420701) as described in Intra-cytoplasmic cytokine staining of IL-6. Cells were fixed, permeabilized, and subjected to intracellular IFN-γ staining with the Cytofix/Cytoperm kit (BD Biosciences, Cat. BD 554714).

### Statistical analysis

Data were statistically analyzed using GraphPad Prism software. Student’s two-tailed unpaired t-test was used to determine the significance of the difference between the means of two groups. One-way ANOVA tests were used to compare the means among three or more independent groups/categories. Data are shown as mean ± SEM. Asterisks indicate significant differences. Significance was set to p < 0.05 (*p < 0.05, **p < 0.01 and ***p < 0.001, ****p < 0.0001).

## Results

### ICAMs are largely dispensable for leukocyte generation and margination inside the lung microvasculature

**Figure 1:**
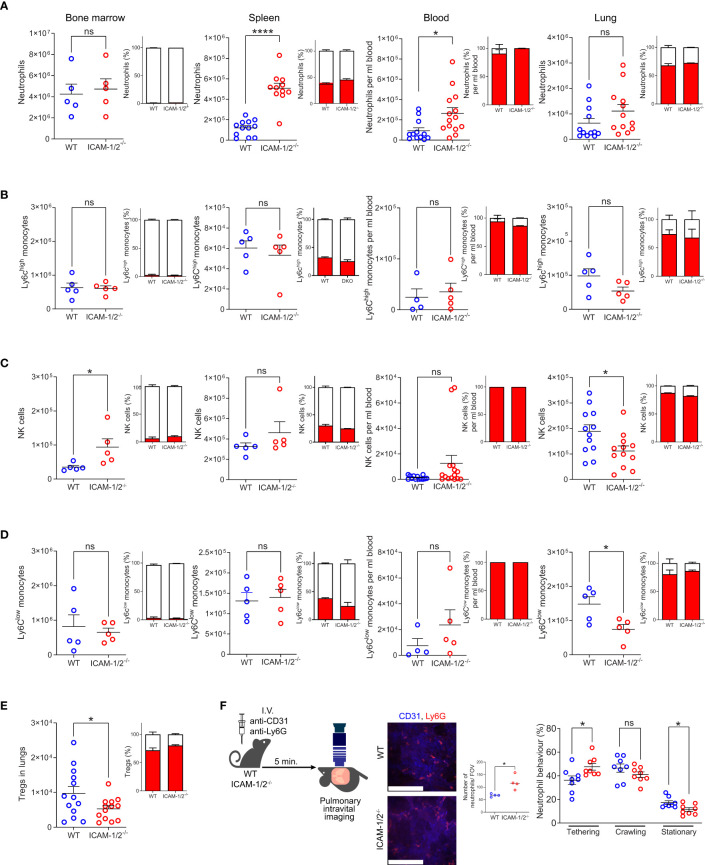
Leukocyte counts, their vascular partition, and neutrophil margination in the lung vasculature are not affected by loss of ICAM-1 and ICAM-2. The relative numbers of neutrophils (CD45^+^CD11b^+^Ly6G^+^) **(A)**, classical (Ly6C^hi^) monocytes (CD45^+^CD11b^+^Ly6G^-^Ly6C^hi^) **(B)**, NK cells (CD45^+^CD11b^+^NKp46^+^) **(C)**, patrolling (Ly6C^lo^) monocytes (CD45^+^CD11b^+^Ly6G^-^Ly6C^low^) **(D)** and Tregs (CD45^+^CD4^+^CD25^+^Foxp3^+^) **(E)** in the indicated organs. The partition of each leukocyte type in intravascular (red) and extravascular (white) organ compartments is shown in the insets next to each graph. n= 5 for all bone marrow determinations; n= 5-12 for spleen; n= 5-13 for lung determinations of either WT or ICAM-1/2^-/-^ mice and n=12-14 for blood samples analyzed for each leukocyte type. Data were combined from at least 2 independent experiments. **(F)** Pulmonary intravital microscopy of neutrophils marginating in either WT or ICAM-1/2^-/-^ lungs. The experimental design is shown in the left panel. Neutrophils and blood vessels were stained by I.V. injection with the indicated fluorescently labeled mAbs. The middle panel depicts representative still images from two-photon video microscopy movies (scale bars represent 128 μm), and the bar graph shows the total neutrophils per field of view (FOV) as assessed by imaging. The total numbers of circulating and adherent neutrophils and their dynamic phenotypes grouped in three categories (tethering, crawling and arrested (stationary)) are depicted in the right panels. n= 8 for each group. Statistical significance was determined by two-tailed, unpaired Student’s t tests. *P < 0.05, ****P < 0.0001, ns, not significant. The error bars indicate the SEM of each measurement.

We initially characterized the cellular composition of all major types of leukocytes in the lungs of ICAM-1/2^-/-^ mice. The total number of the major sentinel leukocytes, alveolar macrophages as well as lung DCs, were normal in the ICAM-1/2^-/-^ mice (**Supplementary Figures 1A–D**). At steady state, the pulmonary capillary bed contains a large pool of marginated leukocytes which comprise 20-60 fold more neutrophils as well as NK cells and monocytes (20) than in the systemic circulation (21). In spite of a similar number of neutrophils produced in the bone marrow of ICAM-1/2^-/-^ mice (**Figure 1A**), we found higher amounts of blood circulating neutrophils, as well as much more abundant spleen neutrophils in these mice (**Figure 1A**). The higher numbers of these neutrophils could be explained by inability of the blood circulating neutrophils to get retained by ICAM-1 and ICAM-2 expressing capillaries and microvessels outside the lungs (22). Nevertheless, the number of neutrophils recovered from WT and ICAM-1/2^-/-^ lungs were similar (**Figure 1A**). Notably, the majority of these lung neutrophils were intravascular, whereas most spleen and bone marrow neutrophils were extravascular in both WT and ICAM-1/2^-/-^ mice (**Figure 1A**). The loss of both ICAMs also did not affect the numbers of Ly6C^high^ inflammatory monocytes marginating through the lung vasculature and their partition between intravascular and extravascular compartments at steady state conditions (**Figure 1B**). In contrast, NK cells and patrolling monocytes (Ly6C^low^) (23), were significantly reduced in the resting lungs (**Figures 1C, D**) highlighting a key role of the two vascular ICAMs in the retention of these leukocytes inside the lung vasculature. Likewise, the numbers of intravascular Tregs recovered from ICAM-1/2^-/-^ lung vessels were significantly reduced relative to WT lung vessels (**Figure 1E**), in spite of similar totals of conventional lung CD4^+^ and CD8^+^ T cells (**Supplementary Figure 2**). Similar to patrolling monocytes, Tregs express constitutively adhesive LFA-1 (24) and may use constitutively expressed MHC-II/self-peptide complexes within subsets of alveolar capillaries for survival (25). In contrast to these leukocytes, iNKT cells appeared normal in spite of their dramatic loss in the ICAM-1/2^-/-^ liver (**Supplementary Figure 3**). Furthermore, a subset of HSCs populating the lungs was also normally retained in ICAM-1/2^-/-^ lungs consistent with their lack of β2 integrin expression (data not shown). Interestingly, this subset also lacked α4 integrin expression, in contrast to BM HSCs (**Supplementary Figure 4**).

To assess whether marginating neutrophils use the capillary expressed ICAM-1 and ICAM-2 for arrest or crawling, we next assessed by intravital microscopy the ability of individual neutrophils to tether (i.e., transient adhesion), continuously crawl or firmly arrest (i.e., stationery) along either WT or ICAM-1/2^-/-^ pulmonary capillaries. Notably, about 50% of neutrophils entering WT capillaries crawled significant distances on these vessels, but this crawling behavior was only mildly reduced in ICAM-1/2^-/-^ lung capillaries (**Figure 1F**, middle bar graph). In contrast, the number of neutrophils firmly arrested (i.e., stationary) inside the capillaries was significantly reduced (**Figure 1F**, right bar graph). Thus, the combined fraction of crawling and firmly arrested neutrophils visualized was slightly but significantly reduced in ICAM-1/2^-/-^ mice. In contrast, deficiency of the CD11b integrin on similar neutrophils totally abrogated their crawling behavior (17). Furthermore, the presence of ICAMs inside all lung vessels as well as on alveolar epithelial cells was dispensable for neutrophil recruitment, extravasation and entry into the bronchoalveolar space during an acute infection with *Pseudomonas aeruginosa*, as well as for bacterial clearance (**Supplementary Figure 5**). Taken together our results suggest that vascular and epithelial ICAMs are dispensable for lung homeostasis and the survival of all major sentinel leukocytes, as well as for the margination of major blood circulating leukocytes except for patrolling monocytes, NK cells and Tregs passing through the pulmonary vasculature.

### ICAMs in the lungs and lung draining lymph nodes are dispensable for influenza clearance, mice recovery and protection from homosubtypic influenza re-infection

**Figure 2:**
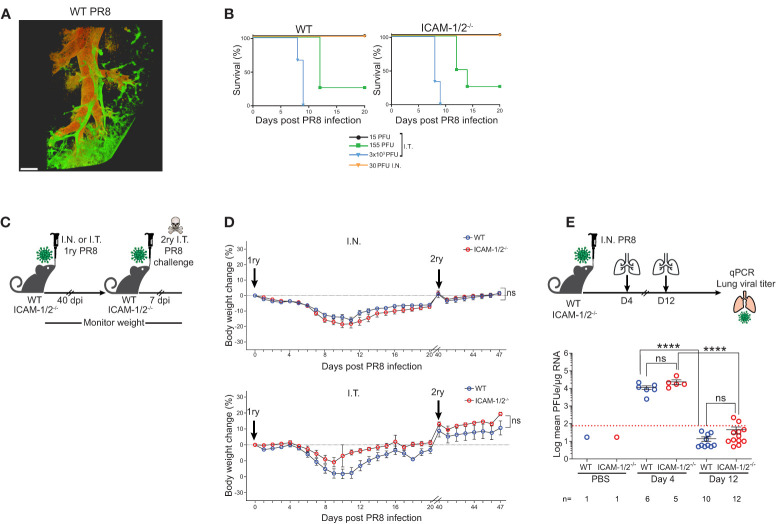
ICAM-1/2^-/-^ mice infected with PR8 exhibit a similar disease progression and viral clearance compared to WT mice. **(A)** Pulmonary imaging of infected mice using three-dimensional (3D) light sheet fluorescence microscopy (LSM). WT mice were I.T. instilled with 3x10^3^ PFU of PR8-mCherry influenza virus. Four days post infection (dpi), mice were sacrificed, lungs were cleared and individual lobes were imaged using an UltraMicroscope II (LaVision BioTec). The distribution of the PR8-mCherry influenza virus was mainly detected in the bronchi and bronchioles. Scale bar represents 500 μm. n=2. **(B)** Kaplan-Meier survival curves of WT (left panel) and ICAM-1/2^-/-^ (right panel) mice infected I.T. or I.N. with the indicated doses of PR8 influenza (15 PFU n=5, 155 PFU n=4, 3x10^3^ PFU n=3 (I.T.), 30 PFU n=4 (I.N.)) were assessed in at least two independent experiments. **(C)** Experimental design and timeline of primary and secondary infections. **(D)** Body weight change in either WT or ICAM-1/2^-/-^ mice initially infected I.N. with 30 PFU (n=13 for each group, top panel) or I.T. with 15 PFU (n=8 for WT and n=6 for ICAM-1/2^-/-^, bottom panel) (1ry), in at least two independent experiments. For a secondary challenge, both groups were challenged I.T. with a lethal dose (3x10^3^ PFU) of PR8 influenza on day 40 (2ry). **(E)** Experimental design and timeline of viral titers in lungs. Mice were infected I.N. with 30 PFU of PR8 influenza, lungs were harvested on day 4 and 12 post infection and the virus lung titer was determined for both mice groups using RT-qPCR. I.N. PBS administration was performed in control experiments. Red dotted line represents the detection limit. **(D)** Statistical analysis was performed using a one-way ANOVA test comparing % body weight loss between groups. **(E)** Statistical significance was determined by two-tailed, unpaired Student’s t tests. ****P < 0.0001, ns, not significant. The error bars indicate the SEM of each measurement.

A classical model of viral lung infections is influenza, a leading cause of respiratory tract diseases worldwide (26). To test the involvement of lung ICAMs in leukocyte recruitment and clearance of this pathogen, we used the lab adapted influenza A virus (A/Puerto Rico/8/1934 (H1N1) (herein: PR8 influenza) (27). We compared two routes of lung infections: the physiological intranasal route (I.N.) and the widely used experimental route of intratracheal infection (I.T.). WT and ICAM-1/2^-/-^ mice were infected with different doses of the PR8 influenza virus and both their weight loss and survival rates were compared. As expected, this virus spread primarily along the bronchial tree of the lungs (**Figure 2A** and **Movie 1**). Strikingly, the mortality rates caused by either semi-lethal or lethal doses of PR8 influenza were indistinguishable between WT and ICAM-1/2^-/-^ mice (**Figure 2B**). Furthermore, similar rates of weight loss were observed in both mice groups in either administration route (I.T. and I.N.) (**Figures 2C, D**). In both cases, by day 12, all mice started to regain weight and by day 40 they returned to or surpassed their original body weight (**Figure 2D**). Consistently with these clinical observations, both PR8 expansion and clearance rates were comparable for both WT and ICAM-1/2^-/-^ mice groups (**Figure 2E**). Since PR8 expands mainly in epithelial cells (2), these results indicate that loss of epithelial ICAMs doesn’t impair influenza infection, contrasting a previous report on ICAM-1 dependence of this infection in airway derived epithelial cells as assessed *in vitro* (28).

The hallmark of normal adaptive response to viral infection is the ability of the infected animals to develop full protection against a secondary challenge with a homosubtypic virus (immunity to the same serotype virus) (29, 30). Both WT and ICAM-1/2^-/-^ mice groups were therefore pre-infected I.N. or I.T. with low doses of PR8 and 40 days later, when full humoral and cellular immunity are established (31), all mice were I.T. challenged with a lethal dose of 3x10^3^ PFU of PR8 (**Figure 2C**). Strikingly, both WT and ICAM-1/2^-/-^ mice similarly survived this secondary lethal virus challenge (**Figure 2D**) suggesting that the ICAM-1/2^-/-^ mice were fully protected from the viral challenge. Thus, ICAM-1/2^-/-^ mice not only normally cleared the PR8 influenza introduced *via* different routes, but also mounted long lasting memory responses against a secondary infection with a lethal dose of a homosubtypic influenza virus challenge.

### ICAMs are dispensable for neutrophil and NK cell entry to influenza infected lungs

**Figure 3:**
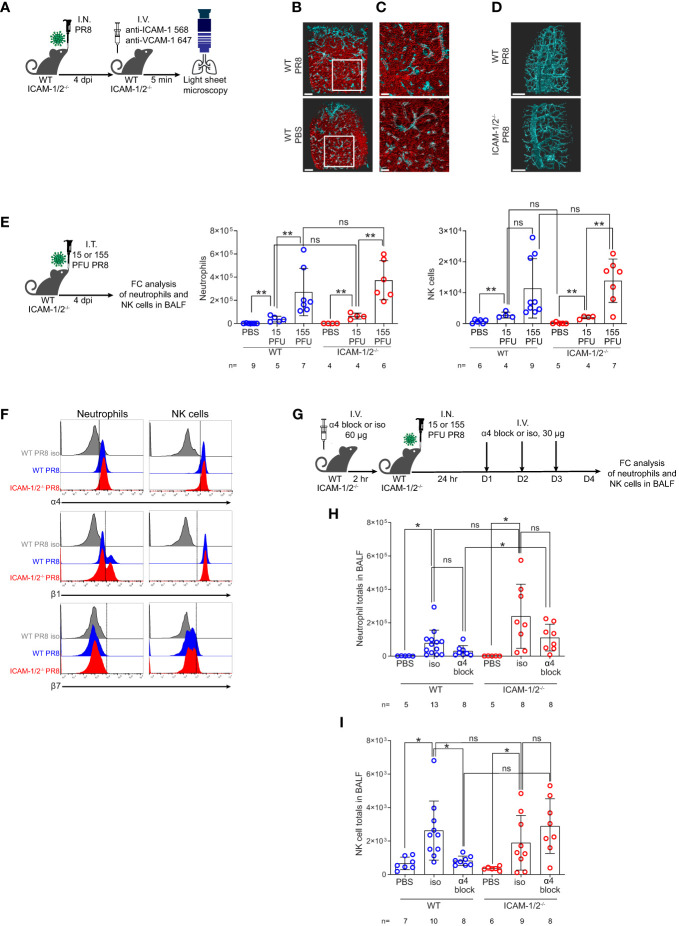
ICAM-1 and VCAM-1 compartmentalization and differential usage by innate leukocytes entering influenza infected lungs. **(A)** Experimental design and timeline of pulmonary imaging by three-dimensional (3D) light sheet fluorescence microscopy (LSM). WT and ICAM-1/2^-/-^ mice were I.N. infected with a sublethal dose of PR8 influenza virus or administered with PBS. 4 days post infection (dpi), mice were subjected to intravital staining by an intravenous co-injection of Alexa-Flour 568 labeled anti ICAM-1 mAb (red) and Alexa-Flour 647 labeled anti VCAM-1 mAb (cyan) **(B, C)**. Scale bars represent 500 μm or 200 μm, respectively. **(B)** The lungs of resting and PR8 infected WT mice were compared by the intravital staining described in **(A)**. **(C)** Enlarged views of the white squares shown in **(B)**. **(D)** WT and ICAM-1/2^-/-^ mice were compared by similar intravital staining as in **(B, C)** with Alexa-Flour 647 anti-VCAM-1 mAb (cyan). Scale bars represent 500 μm. **(E)** Experimental design and timeline (left panel) for determination of neutrophil (middle panel) and NK cell counts (right panel) in the BALF of either WT or ICAM-1/2^-/-^ mice 4 days post I.T. infection with the indicated doses of PR8 influenza. **(F)** Flow cytometry analysis of α4 (top), β1 (middle), and β7 (bottom) integrin expression on neutrophils (left column) and NK cells (right column). **(G)** Experimental design and timeline protocol for α4 blocking in WT and ICAM-1/2^-/-^ mice infected with PR8 influenza. Mice were I.V. administered with 60 ug of anti α4 mAb (α4 block) or α4 isotype control (iso) and 2 hours later were I.N. infected with 30 PFU of PR8 influenza. For the following 3 days, 30 ug anti-α4 blocking or isotype control mAb were I.V. administered once a day. On day 4 post infection, flow cytometry analysis was performed to determine the total number of neutrophils **(H)** and NK cells **(I)** recovered in the BALF of the infected WT or ICAM-1/2^-/-^ mice. I.N. PBS administration was performed in control experiments. Statistical significance was determined by two-tailed, unpaired Student’s t tests. *P < 0.05, **P < 0.01, ns, not significant. The numbers of each experimental group are indicated in the graphs. The error bars indicate the SEM of each measurement.

Initial clearance of influenza A virus infection involves neutrophils and NK cell activities (32, 33). Neutrophils are among the first cells recruited to the lungs in response to this infection, where they phagocytose the virus and initiate various antiviral programs (34, 35). NK cells co-recruited to the infected lungs facilitate elimination of influenza by binding viral HA *via* their NKp44 and NKp46 receptors (36). We therefore next addressed the roles of ICAMs in the recruitment of these leukocytes to PR8 infected lungs during this time frame. Since PR8 infection was restricted to the bronchial tree during the first 4 days of infection (**Figure 2A** and **Movie 1**), we first assessed the luminal distribution of these ligands in different lung vessels around the bronchial tree and in pulmonary capillaries, the two major vascular platforms for leukocyte extravasation into infected lungs (2). To that end, we used LSM microscopy for whole lung imaging of both peribronchial vessels and pulmonary capillaries following intravital luminal staining with fluorescently labeled anti- ICAM-1 and VCAM-1 specific mAbs (**Figure 3A**). Notably, while alveolar capillaries in influenza-infected lungs lacked VCAM-1 expression, high surface ICAM-1 expression was detected on all capillaries (**Figures 3B, C** and **Movies 2**, **3**). In contrast, high and uniform luminal VCAM-1 expression was detected on both large and small bronchi and bronchioles (**Figure 3D** top panel, **Movie 4**). Surprisingly, PR8 infection did not significantly elevate the luminal expression of either ICAM-1 or VCAM-1 in either the peribronchial or capillary lung vasculature (**Figures 3B, C**). Furthermore, VCAM-1 expression on infected peribronchial vessels was not elevated in ICAM-1/2^-/-^ lungs compared to WT lungs, ruling out a compensatory contribution of this ligand to leukocyte entry into the infected ICAM-1/2^-/-^ lungs (**Figure 3D** bottom panel and **Movie 5**). Thus, during infection, the main vascular ligand expressed by the vast majority of peribronchial vessels is VCAM-1, while the main vascular ligand expressed by all pulmonary capillaries is ICAM-1 with constitutive expression of the low affinity LFA-1 ligand ICAM-2 shared by both capillary and peribronchial blood vessels (22).

Assessing the contribution of these ligands to the entry and accumulation of either neutrophils or NK cells in lungs infected with different sublethal viral doses, we found that neither leukocyte used ICAM-1 or ICAM-2 for recruitment into the infected lungs (**Figure 3E**). In order to follow the potential role of VCAM-1 in neutrophil and NK cell recruitment to PR8 infected lungs, we first verified that both leukocytes comparably expressed the main VCAM-1 binding integrin VLA-4 (α4β1) on their surface during active infection in both WT and ICAM-1/2^-/-^ mice (**Figure 3F**). Notably, the majority of the α4 integrin subunit expressed by these leukocytes was associated with β1 rather than with β7 subunits (**Figure 3F**). Interestingly, the introduction of α4 blocking mAb prior and during early phases of infection (**Figure 3G**) did not inhibit neutrophil recruitment to infected lungs of either WT or ICAM-1/2^-/-^ mice in a statistically significant manner (**Figure 3H**). Surprisingly, similar introduction of α4 integrin blocking mAb resulted in significant inhibition of the recruitment of NK cells to PR8 infected WT lungs, consistent with NK cell usage of VCAM-1 for their recruitment (**Figure 3I**). NK cell recruitment to ICAM-1/2^-/-^ lungs, on the other hand, was not inhibited by similar α4 integrin blocking (**Figure 3I**). Importantly, this mAb was not reported to exert cytopathic effects in various chronic inflammatory settings (37). Taken together, neither neutrophils nor NK cells required ICAMs to get recruited into PR8 infected lungs. Since infected lungs do not express the alternative α4 integrin ligand MadCAM-1 (38) and NK cells express negligible α4β7 (**Figure 3F**), our results highlight a potential role of VLA-4-mediated NK cell interactions with VCAM-1 expressed by the peribronchial lung vessels during their entry into influenza infected WT lungs. Neutrophils, on the other hand, enter PR8 infected lungs independently of expression of either capillary expressed ICAMs or peribronchial vessel-expressed VCAM-1 (**Figure 3H**).

### ICAMs are dispensable for CD4^+^ T cell differentiation, Tfh generation, T-dependent B lymphocyte differentiation, and virus specific IgG production following PR8 infection

**Figure 4:**
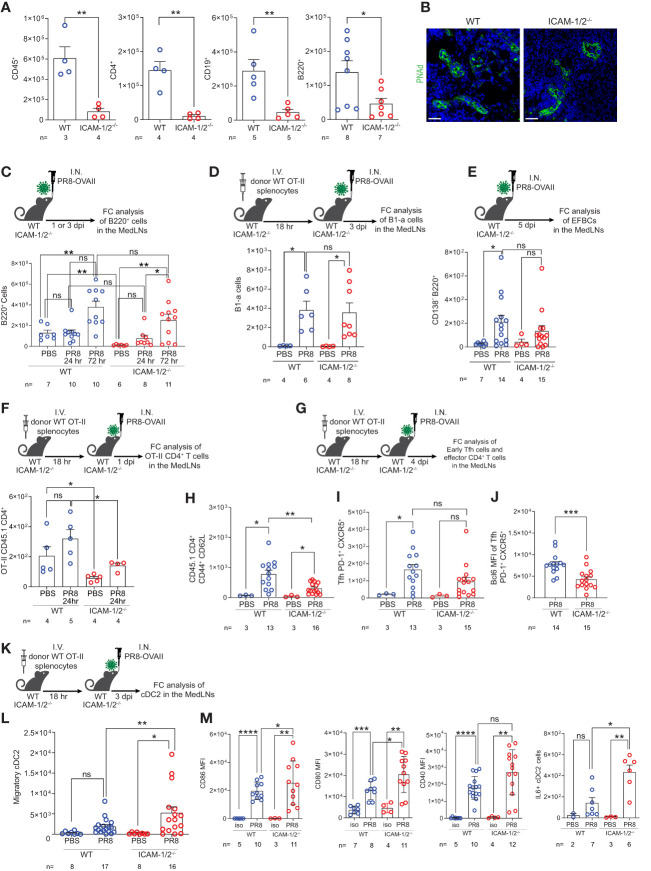
Defective homing of early CD4^+^ T cells post influenza infection is counterbalanced by augmented cDC2 accumulation and activation inside ICAM-1/2^-/-^ mediastinal LNs. **(A)** The total numbers of CD45^+^ cells, CD4^+^ T cells, CD19^+^ and B220^+^ B cells in resting MedLNs of either WT or ICAM-1/2^-/-^ mice determined by flow cytometry. The subset totals were recovered from the same mice, except for the B220^+^ totals, which were analyzed from a separate experiment. **(B)** Paraffin sections of resting MedLNs of naïve WT (left panel) and ICAM-1/2^-/-^ (right panel) mice stained with anti PNAd (green) mAb. Scale bar represents 50µm. **(C)** Experimental design and timeline of B220^+^ B cell accumulation in the MedLNs of ICAM-1/2^-/-^ mice after 1 or 3 days post I.N. infection (dpi) with a sublethal dose of PR8-OVAII influenza. I.N. PBS administration was performed in control experiments. Results shown (bottom panel) are the mean +- SEM. **(D)** Experimental design and timeline of B1-a cell accumulation in the MedLNs of both WT and ICAM-1/2^-/-^ following PR8 infection (top panel). Results shown (bottom panel) are the mean +- SEM. **(E)** Experimental design, timeline and results of accumulation of extrafollicular B cell (EFBC, CD138^+^, B220^+^) in the MedLNs of either WT or ICAM-1/2^-/-^ infected mice. Mice were I.N. administered with a sublethal dose of PR8-OVAII influenza 1 or 3 days post I.N. infection (dpi) (top panel). Results shown (bottom panel) are the mean +- SEM. **(F)** Experimental design and timeline of flow cytometry analysis of OT-II CD45.1 CD4^+^ T cell accumulation at steady state or 1 day following OVA-II PR8 infection (top panel). Results shown are the mean +- SEM (bottom panel). **(G)** Experimental design and timeline of flow cytometric analysis of total OT-II CD45.1 cells and their differentiated derivatives. Numbers of total OT-II CD45.1 cells **(H)**, early Tfh cells (CD45.1^+^PD-1^+^CXCR5^+^) **(I)** and the BCL6 expression levels of these early Tfh cells **(J)** determined in the MedLNs of WT or ICAM-1/2^-/-^ 4 day post OVA-II PR8 infection. **(K)** Experimental design and timeline protocol for the flow cytometry analysis of cDC2s (CD45^+^MHC-II^+^CD11b^+^CD103^-^) accumulated in the MedLNs of WT and ICAM-1/2^-/-^ at steady state or 3 days post infection with OVA-II PR8 (top panel). **(L)** Results shown (bottom left panel) are the mean +- SEM. **(M)** cDC2 activation profiles assessed by expression levels of CD86, CD80, CD40 and IL-6. Statistical significance was determined by two-tailed, unpaired Student’s t tests. *P < 0.05, **P < 0.01, ***P < 0.001, ****P < 0.0001, ns, not significant. The numbers of each experimental group are indicated in the graphs. The error bars indicate the SEM of each measurement.

The MedLNs are the key lung-draining LNs of the lower respiratory tract (39). The early anti-influenza virus clearance is tightly dependent on generation of virus specific IgG antibodies (40) produced by plasma cells in B cell follicles of the MedLNs (41, 42). To further explore the cellular basis for the normal humoral response of ICAM-1/2^-/-^ mice, reflected by normal recovery from sublethal PR8 infection (**Figure 2B**), normal viral clearance (**Figure 2E**), and full protection from a lethal homosubtypic viral challenge 40 days post primary infection (**Figure 2D**), we next dissected the accumulation of individual B and T cell subsets involved in antiviral humoral immunity in MedLNs that drain influenza infected lungs. At steady state, the hematopoietic lineage cells (CD45^+^) populating these LNs including CD19^+^ and B220^+^ B cells as well as CD4^+^ T cells poorly accumulated in the resting MedLNs of ICAM-1/2^-/-^ mice (**Figure 4A**), in spite of normal HEVs in these mice (**Figure 4B**). Strikingly, however, ICAMs were not required for endogenous B-2 cell accumulation in the MedLNs following viral infection (**Figure 4C**). Furthermore, the CD5^+^ B-1a cell subset, which produces natural broad specificity anti-influenza IgMs that contributes to early protection (43), also normally accumulated in ICAM-1/2^-/-^ MedLNs of PR8 infected mice (**Figure 4D**). Similarly, extrafollicular IgG producing plasma cells which provide an initial burst of T-dependent anti-influenza antibodies essential for early control of infection (44), were also recovered at similar numbers in the MedLNs of both WT and ICAM-1/2^-/-^ mice following infection (**Figure 4E** and **Supplementary Figure 6**).

Since B-2 B cells undergo isotype switching and affinity maturation depending on helper viral antigen specific CD4^+^ T cells, we next followed how influenza antigen specific CD4^+^ T cells accumulate inside the lung draining MedLNs of virus infected ICAM-1/2^-/-^ mice. To that end, we infected either WT or ICAM-1/2^-/-^ mice with a PR8 strain encoding the OVAII sequence (OVA_323–339_ peptide) recognized by OT-II CD4^+^ transgenic T cells. As expected, prior to and shortly after infection the homing of transferred virus specific OT-II CD4^+^ T cells into ICAM-1/2^-/-^ MedLNs was markedly impaired (**Figure 4F**). Consistent with their poor homing, the OVA specific CD4^+^ T cells also gave rise to significantly lower numbers of monoclonal OT-II effector CD4^+^ T cells (i.e., CD44^hi^CD62^lo^) by day 4 post infection (**Figures 4G, H**). Strikingly, however, similar numbers of early OT-II T follicular helper (Tfh) cells were recovered inside the MedLNs of both influenza infected WT and ICAM-1/2^-/-^ mice at later time points post infection (**Figure 4I**). Nevertheless, these early Tfh cells expressed lower content of the major Tfh transcription factor, BCL6 (**Figure 4J**), suggesting that their early maturation was partially ICAM-1 and ICAM-2 dependent, possibly due to loss of B cell ICAMs implicated in T-B cell synapses (45).

The differentiation of viral specific naïve CD4^+^ T cells into early and late B cell helper T cells and Tfh effectors, in the MedLNs, depends on proper education by migratory cDC2 subsets carrying influenza antigens into these LNs (46–48). Importantly, at steady state, the cellularity of all cDCs populating the MedLNs remained normal in ICAM-1/2^-/-^ mice (**Supplementary Figure 7**). Nevertheless, the cDC2 subset was recruited at higher numbers to ICAM-1/2^-/-^ LNs following PR8 infection (**Figures 4K, L**), and were hyperactivated, as evident by their significantly elevated levels of the major co-stimulatory molecules CD80 and CD86, as well as elevated IL-6 production (**Figure 4M**). Thus, the proliferation and differentiation of CD4^+^ T cells into effective Tfh lymphocytes was probably driven by DC overexpression of these critical Tfh differentiation cytokines and co-stimulatory molecules. Importantly, the cDC2 hyperactivation and their enriched IL-6 content in the MedLN of infected ICAM-1/2^-/-^ mice did not appear to be the result of the reduced Treg content in the lungs and MedLNs of these mice (**Figure 1E** and **Supplementary Figure 8A**). This conclusion arose from the finding that forced depletion of endogenous Tregs from WT mice with anti-CD25 mAb (**Supplementary Figure 8B**) did not increase cDC2 accumulation or stimulation following PR8 infection (**Supplementary Figures 8C–E**).

**Figure 5:**
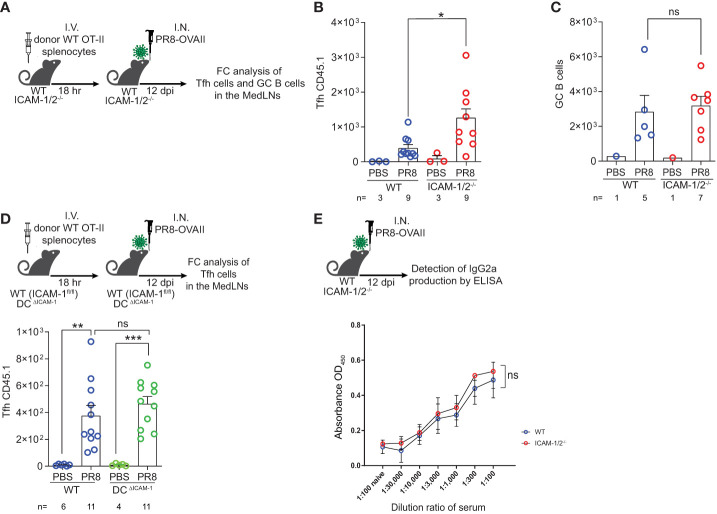
Augmented Tfh differentiation and normal anti-influenza IgG levels in ICAM-1 and ICAM-2 deficient mediastinal lymph nodes. **(A)** Experimental design and timeline of Tfh cell generation and GC B cell accumulation post PR8-OVAII influenza infection. Donor WT CD45.1 OT-II splenocytes (10^5^) were I.V. transferred into WT and ICAM-1/2^-/-^ CD45.2 mice and mice were I.N. infected with a sublethal dose of PR8-OVAII influenza virus. 12 days post infection (dpi), MedLNs were harvested and the total number of **(B)** OT-II Tfh cells (CD4^+^CD44^+^CD62L^-^PD-1^+^CXCR5^+^) and **(C)** GC B cells (B220^+^CD138^-^CD38^-^Fas^+^) were determined in the indicated MedLNs. I.N. PBS administration was performed in control experiments. **(D)** Experimental design and timeline for the flow cytometry analysis of OT-II Tfh cells (CD4^+^CD44^+^CD62L^-^PD-1^+^CXCR5^+^) generated 12 days post infection in either WT (ICAM-1^fl/fl^) or DC^ΔICAM-1^ mice (top panel). Results shown (bottom panel) are the mean +- SEM. **(E)** Experimental design and timeline of the detection of PR8 specific IgG2a levels in the sera of PR8 infected mice (top panel). Sera were collected on day 12 post infection and the levels of PR8 specific IgG2a were determined in triplicates. Results shown (bottom panel) are the mean +- SEM. n= 3 for each group. Statistical significance was determined by two-tailed, unpaired Student’s t tests. *P < 0.05, **P < 0.01, ***P < 0.001, ns, not significant. The numbers of each experimental groups are indicated in the graphs. The error bars indicate the SEM of each measurement.

We next followed the fate of monoclonal Tfh cells on day 12 post infection, when germinal centers (GCs) are generated inside the MedLNs (**Figure 5A**). Strikingly, significantly elevated numbers of monoclonal OVA specific Tfh cells were generated from adoptively transferred naïve CD4^+^ OT-II T cells inside MedLNs of influenza infected ICAM-1/2^-/-^ mice (**Figure 5B**). In contrast, the total numbers of endogenous polyclonal GC B cells (Fas^+^, CD38^low^) recovered 12 days post infection were similar in WT and ICAM-1/2^-/-^ MedLNs (**Figure 5C**). To dissect the potential contribution of DC ICAMs to this multistep humoral response separately from the roles of endothelial and stromal ICAMs, we prepared a new mouse model devoid of DC ICAM-1 by first generating a new strain of ICAM-1^fl/fl^ mice and then crossing them with mice expressing the Cre recombinase under the DC enriched CD11c promoter (herein DC-specific ICAM-1^-/-^ or DC^ΔICAM-1^ mice), (**Supplementary Figures 9A, B**). These mice lost ICAM-1 expression on all major cDC subsets and moDCs in the MedLNs, but retained normal ICAM-1 expression by pDCs, accessory IFN-α producing DCs involved in Th1 and Tc1 differentiation processes (49), as well as by subcapsular sinus macrophages and B cells (**Supplementary Figure 9B**). Importantly, the negligible ICAM-2 levels expressed by cDCs were not elevated in our DC^ΔICAM-1^ mice (**Supplementary Figure 9C**). Notably, the ability of adoptively transferred naïve OT-II CD4^+^ T cells to accumulate and differentiate into GC Tfh cells was normal in the DC^ΔICAM-1^ mice (**Figure 5D**) as opposed to their enhanced differentiation in the total ICAM knockout (ICAM-1/2^-/-^) MedLNs (**Figure 5B**). Collectively, these results suggest that in the absence of stromal ICAM expression (endothelial, fibroblastic and B cell), CD4^+^ effector T cells gave rise to elevated numbers of long lasting highly effective Tfh lymphocytes. These T cells provided critical help for the viral Ag-specific ICAM-1/2^-/-^ B-2 cells that entered the ICAM deficient MedLNs in response to viral infection. Consequently, the ICAM-1/2^-/-^ influenza specific plasmablasts, produced normal serum levels of anti-PR8 IgGs after both primary infection and secondary challenge (**Supplementary Figure 10**). The levels of the main influenza clearing IgG subtype IgG2a (50), and the PR8 binding affinities of these polyclonal Abs recovered from the blood of ICAM-1/2^-/-^ mice determined at high sera dilutions, were also normal (**Figure 5E**). These surprising results suggest that T-dependent B cell immunity against the PR8 influenza virus does not require DC or B cell ICAM-1 and ICAM-2 expression, but is fine-tuned by stromal ICAMs, possibly expressed by fibroblastic reticular cells (FRCs) and follicular dendritic cells (FDCs). Thus, all major components of anti-viral humoral immunity are normally elicited in lung draining LNs deficient in ICAM-1 and ICAM-2 during a primary influenza infection, resulting in normal IgG dependent viral clearance.

### CD8^+^ lymphocytes use ICAMs to enter MedLNs during influenza infection but undergo robust virus dependent differentiation into effectors in the absence of stromal and DC ICAMs

**Figure 6:**
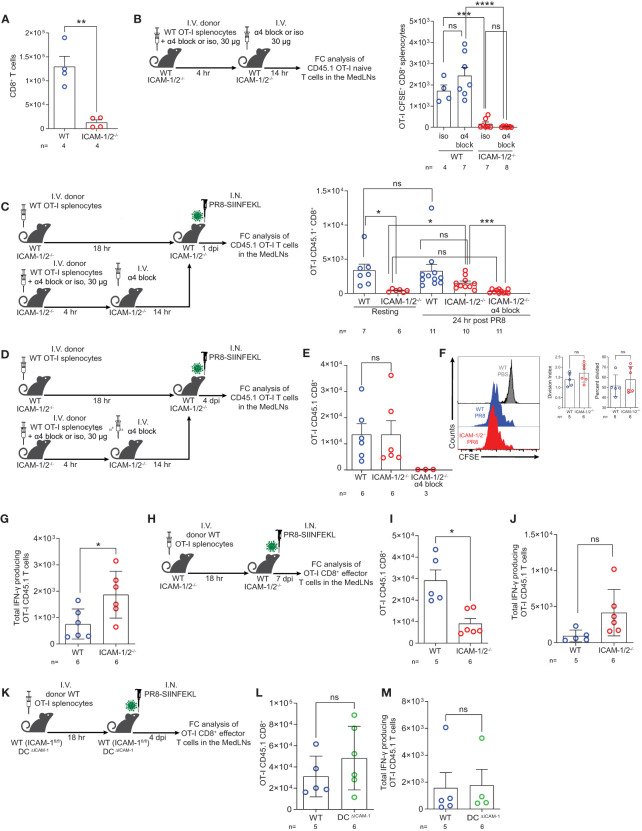
Naïve CD8^+^ lymphocytes vigorously proliferate and differentiate in the mediastinal LNs of influenza infected mice in the absence of ICAM-1 and ICAM-2 and independently of DC ICAM-1. **(A)** The total numbers of CD45^+^CD8^+^ T cells in the resting MedLNs of both WT and ICAM-1/2^-/-^ mice analyzed by flow cytometry. **(B)** Experimental design and timeline protocol for OT-I splenocyte homing to resting MedLNs of WT and ICAM-1/2^-/-^ mice. Naïve CD45.1 OT-I (2x10^6^) were preincubated with α4 mAb (α4 block) or isotype control (iso) and then I.V. transferred into WT and ICAM-1/2^-/-^ mice. An additional dose of α4 blocking mAb or an isotype control mAb was I.V. administered 4 hours later. MedLNs were harvested 18 hours after the initial cell transfer and the accumulation of naïve OT-I (CD45.1^+^CD8^+^CD62L^+^CD44^-^) T cells was determined by flow cytometry. Results shown (right panel) are the mean +- SEM. **(C)** Experimental design and timeline of accumulation of CD45.1 OT-I CD8^+^ T cells in the MedLNs of resting or PR8-SIINFEKL infected WT and ICAM-1/2^-/-^ mice. The OT-I totals in the MedLNs of resting or infected WT or ICAM-1/2^-/-^ mice are shown (right panel). Where indicated, OT-I T cells were preincubated with α4 mAb (α4 block) and recipient mice were treated with an additional dose of α4 blocking mAb 4 hours later as in **(B)**. I.N. PBS administration was performed in control experiments. **(D)** Experimental design and timeline of the proliferation and differentiation of transferred OT-I spenocytes into IFN-γ producing OT-I effector cells in the MedLNs of PR8-SIINFEKL infected WT or ICAM-1/2^-/-^ mice. A total of 2x10^6^ CFSE-labeled CD45.1 OT-I cells were I.V. transferred into both mice groups. After 18 hours, mice were I.N. infected with a sublethal dose of PR8-SIINFEKL. **(E)** Four days post infection MedLNs were harvested and the numbers of effector (CFSE-labeled CD45.1^+^CD8^+^CD62L^-^CD44^+^) OT-I T cells were determined by flow cytometry. Where indicated, OT-I T cells were treated with α4 mAb (α4 block) as in **(C)**. **(F)** Proliferation histograms of the CFSE-labeled CD45.1^+^CD8^+^CD62L^-^CD44^+^ OT-I effector T cells recovered from WT or ICAM-1/2^-/-^ MedLNs post infection described in **(E)** or under steady state (PBS). Inset: The percentage of effector OT-I CD8^+^ T cells that divided at least once and their division index determined four days post PR8-SIINFEKL infection. **(G)** The number of OT-I effector cells described in **(E)** with high IFN-γ content. **(H)** Experimental design and timeline of the proliferation and differentiation in the MedLNs, of OT-I CD8^+^ T cells 7 days post PR8-SIINFEKL infection of WT or ICAM-1/2^-/-^ mice. The numbers of OT-I CD8^+^ effector T cells and of high IFN-γ producing T cells harvested 7 days post infection in the MedLN are shown in **(I, J)**, respectively. **(K)** Experimental design and timeline of the proliferation and differentiation in the MedLNs, of OT-I CD8^+^ T cells 4 days post PR8-SIINFEKL infection of WT (ICAM-1^fl/fl^) or DC^ΔICAM-1^ mice. The numbers of OT-I CD8^+^ effector T cells **(L)** and of high IFN-γ producing T cells **(M)**. Statistical significance was determined by two-tailed, unpaired Student’s t tests. *P < 0.05, **P < 0.01, ***P < 0.001, ****P < 0.0001, ns, not significant. The numbers of each experimental group are indicated in the graphs. The error bars indicate the SEM of each measurement.

Virus specific CD8^+^ T cells contribute to efficient viral clearance by killing of virus infected cells and later on, generate tissue resident memory (T_RM_) cells that can rapidly respond to a secondary heterosubtypic viral infection (51, 52). In light of the enhanced differentiation of CD4^+^ T cells into Tfh cells taking place in PR8 infected ICAM-1/2^-/-^ MedLNs (**Figure 5B**), we next asked whether virus specific naïve CD8^+^ T cells also undergo enhanced differentiation into virus specific CD8^+^ T effectors and T_RM_ CD8^+^ cells following infection with PR8-SIINFEKL (OVA_257–264_ peptide). As expected, the number of endogenous naïve CD8^+^ T cells harvested from ICAM-1/2^-/-^ MedLNs was significantly reduced compared with WT MedLNs (**Figure 6A**). Furthermore, and reminiscent of the defective OT-II CD4^+^ T cell homing into MedLNs of ICAM-1/2^-/-^ mice, the entry of OT-I CD8^+^ T cells into resting MedLNs of ICAM-1/2^-/-^ mice was also markedly reduced compared to WT MedLNs (**Figures 6B, C**). Although OT-I CD8^+^ T cells express comparable levels of the α4 integrins VLA-4 and α4β7 (**Supplementary Figure 11**) and HEVs of the MedLNs express both MadCAM-1 (53) and VCAM-1 (data not shown), under resting conditions, α4 integrin blocking of these T cells had no effect on their homing to either WT or to ICAM-1/2^-/-^ MedLNs (**Figure 6B**). Nevertheless, following influenza infection, the homing of OT-I CD8^+^ T cells into ICAM-1/2^-/-^ MedLNs was only partially reduced (**Figure 6C**; 3^rd^ *vs*. 4^th^ column), and the residual CD8^+^ OT-I T cell homing observed could be completely blocked by anti α4 integrin mAb treatment (**Figure 6C**; 4^th^ *vs*. 5^th^ column). These results indicated that during infection, naïve CD8^+^ T cells can use both HEV expressed ICAMs and α4 integrin ligands like VCAM-1 and MadCAM-1 to enter MedLNs.

We next examined if viral Ag presenting ICAM deficient DCs can drive the proliferation and differentiation of the few OT-I CD8^+^ T cells accumulated in the ICAM-1/2^-/-^ MedLNs on day 4 p.i (**Figure 6D**). In spite of the lack of ICAMs on all APCs and stromal cells of the MedLNs, the numbers of proliferating OT-I CD8^+^ T cells as well as their division rates were comparable in both WT and ICAM-1/2^-/-^ MedLNs (**Figures 6E, F**). As expected, when OT-I CD8^+^ T cells were blocked from entering the infected ICAM-1/2^-/-^ MedLNs in the presence of α4 blocking mAb (**Figure 6D**), no accumulation and generation of effector OT-I CD8^+^ T cells could be detected on day 4 p.i. (**Figure 6E**; 3^rd^ column). Nevertheless, once entering the ICAM-1/2^-/-^ MedLNs following infection and without α4 blocking, the accumulating OT-I CD8^+^ T cells underwent enhanced differentiation into effector IFN-γ producing CD8^+^ T cells (**Figures 6G–J**) similar to the enhanced CD4^+^ T cell differentiation into Tfh cells observed in infected ICAM-1/2^-/-^ mice (**Figure 5B**). This IFN-γ producing subset could evolve by homotypic interactions of the daughter ICAM-1 expressing OT-I CD8^+^ effector T cells which secrete IFN-γ to each other (54).

In contrast to their altered homing and differentiation patterns in the total ICAM-1/2^-/-^ mice, the number of OT-I CD8^+^ T cells entering and proliferating in the MedLNs of the DC^ΔICAM-1^ mice (**Figures 6K, L**) were similar to those found in the MedLNs of the WT mice (**Figure 6E**). Furthermore, the differentiation of the adoptively transferred naïve OT-I CD8^+^ T cells into IFNγ^+^ effector T cells was not affected by ICAM-1 deficiency on all MedLN cDCs in the DC^ΔICAM-1^ mice (**Figure 6M**). Collectively, these results indicated that DC ICAM-1 is not required for normal CD8^+^ T cell priming and differentiation triggered by influenza expressed antigens (**Figures 6L, M**). Since ICAM-1 deficient DCs also lacked ICAM-2 expression (**Supplementary 9C**) these results further suggest that the presence of ICAMs on these essential APCs is dispensable for normal naïve CD8^+^ T cell priming and differentiation in the course of primary influenza lung infection.

### Effector virus specific CTLs accumulate at reduced numbers in infected ICAM-1 and ICAM-2 deficient lungs but give rise to normal T_RM_ CD8^+^ cells that protect from homo- and hetero- subtypic infections

**Figure 7:**
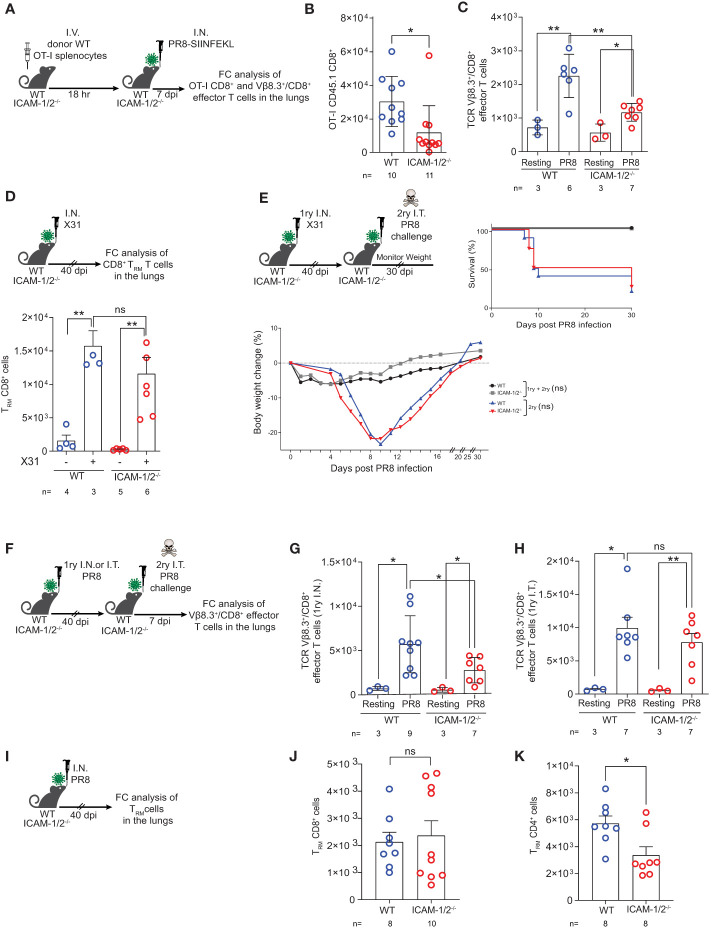
Virus specific CTLs poorly accumulate in infected ICAM-1- and ICAM-2- deficient lungs but give rise to normal T_RM_ CD8^+^ cells that protect from homo- and hetero- subtypic infections. **(A)** Experimental design and timeline of the accumulation of OT-I CD8^+^ effector T cells in the lungs of either WT or ICAM-1/2^-/-^ infected mice. A total of 2x10^6^ CD45.1 OT-I splenocytes were I.V. transferred into WT or ICAM-1/2^-/-^ mice and 18 hours later, mice were I.N. infected with a sublethal dose of PR8-SIINFEKL. **(B)** 7 days post infection, lungs were harvested and the number of CD45.1 OT-I CD8^+^CD62L^-^CD44^+^ effector T cells recovered from the MedLNs were determined by flow cytometry of total lung cell suspensions. **(C)** The totals of endogenous Vβ8.3^+^/CD8^+^ T cells recovered from the PR8-SIINFEKL infected lungs of either WT or ICAM-1/2^-/-^ mice challenged in **(B)** were determined 7 days post PR8-SIINFEKL infection. I.N. PBS administration was performed in a control experiment. **(D)** Experimental design and timeline protocol for primary infection with X31 influenza virus. WT and ICAM-1/2^-/-^ mice were I.N. infected with 10^4^ PFU of X31 influenza or PBS treated. 40 days later, the numbers of T_RM_ CD8^+^ cells (CD62L^lo^CD44^high^CD69^+^CD49a^+^CD103^-/+^) recovered from the lungs of both mice groups were determined (top panel). Results shown (bottom panel) are the mean +- SEM. **(E)** Experimental design and timeline of primary X31 influenza infection (1ry) followed by secondary PR8 influenza challenge (2ry). WT and ICAM-1/2^-/-^ mice were I.N. infected with 10^4^ PFU of X31 influenza. 40 days later, all 4 groups were challenged I.T. with a high dose of PR8 influenza virus (600 PFU) and their weight (bottom panel) and survival (right panel) were monitored for 30 days post-secondary challenge. Mice that lost over 25% of their initial weight were humanly euthanized. **(F)** Experimental design and timeline protocol for primary PR8 infections (1ry) followed by a second challenge (2ry). WT and ICAM-1/2^-/-^ mice were initially infected with 30 PFU (I.N.) or 15 PFU (I.T.) of PR8. 40 days later, both groups were challenged I.T. with a lethal dose (3x10^3^ PFU) of PR8. 7 days later mice were sacrificed and the numbers of endogenous TCR Vβ8.3^+^/CD8^+^ CD62L^-^CD44^+^ T cells were determined in the lungs of both mice groups. **(G)** Results shown from homosubtypic I.N. infection followed by I.T. challenge. **(H)** Results shown from homosubtypic I.T. infection and challenge. PBS administration was performed in control experiments. **(I)** Experimental design for detection of T_RM_ CD8^+^ and CD4^+^ cells generated following primary PR8 infection. WT and ICAM-1/2^-/-^ mice were I.N. infected with 30 PFU of PR8 influenza. 40 days later, the numbers of T_RM_ CD8^+^ cells recovered from total lung cell suspensions were determined and are shown in **(J)**. The numbers of recovered T_RM_ CD4^+^ cells (CD62L^lo^CD44^high^CD69^+^CD49b^+^CD103^-/+^) are shown in **(K)**. **(B–D, G–K)** Statistical significance was determined by two-tailed, unpaired Student’s t tests. **(E)** Statistical analysis was performed using a one-way ANOVA test comparing % body weight loss between groups. *P < 0.05, **P < 0.01, ns, not significant. The numbers of each experimental group are indicated in the graphs. The error bars indicate the SEM of each measurement.

Effector CD8^+^ T cell lymphocytes migrate to the inflamed lung and clear virus infected airways (1) by killing infected epithelial cells (26) and by producing proinflammatory cytokines (e.g., IFN-γ, TNF-α, IL-17 and CCR5 chemokines) that contribute to the recruitment and *in situ* activation of additional inflammatory DCs and pDCs at sites of viral infection (55). To follow the fate of OT-I CD8^+^ T cell effectors generated in the ICAM-1/2^-/-^ lung draining LNs in response to PR8-SIINFEKL infection, we next determined the accumulation of effector OT-I CD8^+^ T cells in the infected lung (**Figure 7A**). Notably, the accumulation of virus Ag specific OT-I CD8^+^ T cells in the ICAM-1/2^-/-^ lungs was significantly reduced compared with WT lungs (**Figure 7B**). The accumulation of polyclonal influenza specific CD8^+^ T cells (i.e., expressing the TCR Vβ8.3 gene segment enriched in CD8^+^ TCRs specific for the immunodominant NP366–374/Db influenza epitope) (56) was also reduced (**Figure 7C**).

Heterosubtypic immunity is defined as cross-protection to infection with an influenza A virus serotype other than the one used for primary infection (29). Effector CD8^+^ T cells receive local instructive signals that promote their subsequent differentiation into T_RM_ cells (57), which are critical for first line protection from a heterosubtypic distinct influenza A virus challenge (58, 59). Successful heterosubtypic responses rely on the presence of these T_RM_ cells which are specific for conserved antigenic moieties shared by non-identical influenza strains (60). In the face of a heterosubtypic influenza A viral infection, the neutralizing antibodies generated by viral-specific B cells, can recognize only the specific influenza A viral surface proteins from a previous infection, while viral-specific CTLs target viral peptides derived from internal proteins of the virus conserved across different subtypes (61). We therefore next followed the fate of polyclonal effector CD8^+^ T cells residing in the ICAM-1/2^-/-^ lungs 40 days post primary infection with the influenza H3N2 subtype A/HK/X31 strain. Strikingly, the number of polyclonal T_RM_ cells generated following X31 influenza infection was similar in ICAM-1/2^-/-^ and WT mice (**Figure 7D**). These results confirmed that long lasting protective T_RM_ CD8^+^ cells are generated independently of ICAM-1 and ICAM-2 (59). Consequently, when ICAM-1/2^-/-^ mice were pre-infected with non-lethal doses of X31 influenza and then subjected to a secondary infection with a high dose of the heterosubstypic PR8 strain 40 days later, the ICAM-1/2^-/-^ mice were fully protected with comparable efficacy to that of WT mice from the heterosubtypic influenza infection (**Figure 7E**). These results indicated that long lasting protective T_RM_ CD8^+^ cells are generated independently of vascular, epithelial or APC ICAMs. These results are also consistent with the lack of ICAM-1 expression by bronchial epithelia (22) and the downregulation of LFA-1 by interstitial T_RM_ CD8^+^ cells at their early stages of differentiation from virus specific effector T cells accumulated early on in the airway mucosa (62).

Finally, we asked whether polyclonal anti-viral Vβ8.3^+^/CD8^+^ T cells can be generated from memory CD8^+^ T cells also following a secondary homosubtypic PR8 influenza challenge of ICAM-1/2^-/-^ mice (**Figure 7F**). Notably, the numbers of Vβ8.3^+^/CD8^+^ effectors recovered in total lung suspensions of I.N. infected lungs subjected to a secondary I.T. homosubtypic PR8 influenza challenge were reduced in ICAM-1/2^-/-^ lungs compared with WT lungs (**Figure 7G**). Nevertheless, the numbers of Vβ8.3^+^/CD8^+^ effectors recovered in total lung suspensions of I.T. infected lungs, subjected to a secondary I.T. homosubtypic PR8 influenza challenge, were higher than in the primary I.N. infection and similar for both WT and ICAM-1/2^-/-^ mice (**Figure 7H**). These results suggest that the primary I.T. and I.N. infection routes induce either ICAM-1 independent or ICAM-1 dependent generation of Vβ8.3^+^/CD8^+^ effectors, respectively, following the recall I.T. infection.

Furthermore, the generation and retention of T_RM_ CD8^+^ cells following I.N. infection (**Figure 7I**) was also normal in ICAM-1/2^-/-^ lungs (**Figure 7J**). In sharp contrast, the number of T_RM_ CD4^+^ cells generated in similarly infected lungs was reduced in ICAM-1/2^-/-^ lungs compared with infected WT lungs (**Figure 7K**). Since this subset was reported to retain its LFA-1 expression (63), it could depend on lung ICAM expression for survival, possibly because these T cells use this integrin to reside in ICAM-1 rich areas like the alveoli. Taken together, our results suggest that although generated at lower rate inside ICAM-1/2^-/-^ MedLN following influenza infection, and poor accumulation of this subset in the infected lungs on day 7 post infection, effector CD8^+^ T cells successfully accumulate in the infected airway mucosa and give rise to T_RM_ CD8^+^ cells (**Figure 5J**), the major resident memory T cells that provide protection from secondary influenza challenges, in particular of heterosubtypic nature.

## Discussion

Influenza virus (flu) is the most common respiratory infectious pathogen (64). Active influenza viruses uses their hemagglutinin (HA) envelope proteins to bind to specific sialic acid residues on epithelial cells in the upper and lower respiratory tract and subsequently gain entry into these cells and replicate (65, 66). We have used the well-studied murine H1N1 influenza A virus to follow the role of ICAMs in this classical model of respiratory viral lung infection. The epithelial cells of the respiratory tract are the main source of productive influenza infection (67). Multiple redundant effector mechanisms of innate and adaptive cells contribute to clearance of this virus and are crucial for the establishment of long lasting immunity against recall infections with identical and related strains (68).

Leukocyte emigration from the blood to the influenza-infected respiratory tract occurs through three major types of blood vessels (1): the postcapillary venules along the bronchial tree (2), the alveolar capillaries in the lung parenchyma and (3) the HEVs of lung draining LNs and tertiary lymphoid tissues that are composed of specialized cuboidal postcapillary venules and serve as the main portal for the entry of circulating naïve and memory T and B lymphocytes into these organs (2). Influenza virus infection is initiated in the trachea and bronchial tree but can spread to alveolar lung regions and cause pneumonia (69). The endothelial lining of the alveolar capillaries wrapped around the numerous alveoli that comprise the lung parenchyma is structurally distinct from that of postcapillary venules (70). The capillary endothelia are elongated and flattened to improve gas exchange (2), demonstrate a lower permeability to solutes and fluids compared to the larger pulmonary blood vessels (70) and have smaller diameters than leukocytes, resulting in slow transit time. Consequently, leukocyte adhesion to and crossing of these blood vessels is drastically different from leukocyte adhesion and emigration from postcapillary venules. Indeed, the classic recruitment paradigm for these lung capillary vessels does not involve selectin-mediated tethering and rolling, and the role of leukocyte integrins is probably far more restricted than in post capillary lung venules (2). These capillaries contain a large number of marginated neutrophils, monocytes, as well as naïve, memory and effector T cells released from infected lung draining LNs (1) The role of integrin ligands, in general and of ICAMs, in particular in this leukocyte margination and extravasation across the lung capillaries has remained vague (4, 17). Our results rule out that influenza infection elevates ICAM-1 expression on alveolar capillaries and suggests that ICAM-1 and ICAM-2 do not serve as the primary ligands for neutrophil margination at steady state conditions. Furthermore, during viral infection, the presence of ICAM-1 or of ICAM-2 on all lung capillaries is also not required for neutrophil or NK cell emigration into the alveolar space. ICAM-1 expression on alveolar epithelial cells is also not critical for leukocyte entry into the bronchoalveolar spaces during this viral infection. Thus, neutrophils localized inside the lung capillaries do not seem to use their LFA-1 integrin to bind endothelial ICAMs. Instead, they may use their Mac-1 integrin for margination through the capillaries (17). Inflammatory monocytes also seem to accumulate in the lung capillaries independently of endothelial ICAMs. In contrast, NK cells, patrolling monocytes and Tregs passing through the lung vasculature seem to use capillary ICAMs, consistent with expression of highly adhesive LFA-1 on the last two (23). It is therefore tempting to speculate that these ICAMs contribute to endothelial repair by patrolling monocytes and Tregs following influenza related injury. In addition, the expression of capillary ICAMs might be critical for the ability of effector T cells to interact with these blood vessels post infection where they encounter critical chemotactic exit signals into infected alveoli (22, 71).

Tracheal and peribronchial postcapillary venules and other blood vessels support massive leukocyte recruitment into the various airways following viral infection (72). In a previous study we found that at steady state peribronchical vessels express high levels of the key VLA-4 integrin ligand VCAM-1 as well as the low affinity LFA-1 integrin ligand ICAM-2 (22). In the present study, using intravital staining with distinct anti VCAM-1 and ICAM-1 mAbs, we have confirmed these results in resting lungs and extended them to influenza infected lungs, where we found a striking compartmentalization of VCAM-1 and ICAM-1 expression by peribronchial *vs*. alveolar capillaries, respectively. Canonical inflammatory cytokines like TNF-α, IL-1β and interferons, are secreted by virus-infected epithelial and stromal cells during various stages of influenza infection (2). Surprisingly, however, we did not find evidence for *de novo* expression of ICAM-1 on the surface of endothelial cells comprising the capillaries or the peribronchial vessels in influenza infected lungs. These staining results suggest that the entry of virus specific effector T cell egressing the MedLNs on their way to the bronchial mucosa during viral infection, likely involves VCAM-1 rather than ICAM-1 and ICAM-2 mediated adhesions. This subset of effector T cells that accumulate nearby the bronchioles and bronchi, once entering the bronchial mucosa, downregulates its LFA-1 within hours (62, 73) and progressively differentiates into T_RM_ cells by elevating CD103 (the integrin receptor for E-cadherin enriched in epithelial cell junctions), CD49a (a collagen type IV receptor) (58) and CD69 that negatively regulates the egress receptor S1P1 (73). Furthermore, in spite of considerable expression of VLA-4 on neutrophils and NK cells the VLA-4-VCAM-1 axis was not mandatory for neutrophil and NK leukocyte entry into similar bronchoalveolar compartments of influenza infected lungs in the absence of ICAMs. Thus, innate leukocyte trafficking through peribronchial blood vessels triggered by viral infection may involve alternative integrin-ligand pairs rather than ICAMs or VCAM-1. In addition, neutrophils and NK cells may emigrate through the pulmonary capillaries of infected alveoli also *via* ICAM-1, ICAM-2 and VCAM-1 independent interactions. One such interaction could involve the β2 integrin Mac-1 shared by neutrophils and NK cells. Mac-1 (CD11b) binds at least 30 alternative ligands in addition to ICAM-1 (74), including endothelial heparan sulfates, JAM-C and ECM ligands that can coat pulmonary vessels like fibrinogen and fibronectin (75). Thus, the direct involvement of Mac-1 in neutrophil and NK cell recruitment to influenza infected lungs remains to be investigated in future studies (76).

HEVs are the third class of specialized blood vessels (77) that drain lymphocytes into the lung draining LNs and into specialized tertiary lymphoid organs along the bronchial tree (78). These vessels serve as the main portal entry for naïve T and B lymphocytes, central memory lymphocytes and subsets of NK and innate lymphoid cells (ILCs) (79). In our influenza infection model, the major posterior MedLNs drain most of the migratory DCs, including the main viral-antigen cross presenting CD103^+^ cDC1 subset (80). In addition, cervical LNs drain parts of the upper airways and are also implicated in virus-specific T cell priming by influenza antigens (81). The LFA-1 integrin ligands ICAM-1 and ICAM-2 are critical for lymphocyte arrest on peripheral LN HEVs (82). Our study confirms the role of these two ligands in T and B cell entry to resting MedLNs but is the first to suggest that during the course of acute influenza infection both types of lymphocytes can use their α4 integrins to enter these LNs in the absence of HEV expressed ICAMs. Indeed, MedLN HEVs constitutively express the VLA-4 ligand VCAM-1 as well as MadCAM-1. The contribution of these ligands to ICAM independent lymphocyte entry is higher following infection, possibly due to upregulation of these ligands. In addition, whereas naïve T and B lymphocytes require the chemokine receptor CCR7 to activate their LFA-1, arrest on HEVs and cross these vessels, following infection, additional signals such as CXCR3 chemokines may provide critical α4 integrin activating signals that allow these lymphocytes to overcome the absence of ICAMs on MedLNs and use their α4 integrins to arrest on HEVs expressed VCAM-1 and MadCAM-1.

Both the cellular and humoral responses critical for influenza clearance depend on the proper presentation of viral antigens by respiratory viral antigen loaded DCs entering the MedLNs (83, 84). The migratory lung CD103^+^ cDC1 subset is the dominant lung DC population to transport influenza virus antigen to the MedLNs from primarily dying respiratory epithelial cells (85). In primary infections, the CD103^+^ cDC1 subset is crucial for efficient processing and presentation of viral antigens to both naïve CD8^+^ and CD4^+^ T cells in these LNs (84, 86). Another migratory DC subset, CD11b^+^ cDC2, is involved mainly in CD4^+^ T cell priming into Th1 and Tfh effector lymphocytes (87). This subset is also capable of transferring viral antigens to resident cDC1 subsets which can in turn present these antigens to CD8^+^ T cells in the MedLNs (88). An asymmetrical distribution of these LN cDC1 and cDC2 subsets create regionalized activation of CD4^+^ and CD8^+^ T cells in the outer cortex and in the paracortex of the LNs, respectively (88). In addition, an influx of CD11b^+^ cDC2 subset can also promote CD8^+^ T cell priming and differentiation (89). The interactions of lymphocyte LFA-1 with ICAM-1 expressed by these DCs, elevated upon infection, maturation and entry into MedLNs, were thought to be critical for functional immune synapses between both CD4^+^ and CD8^+^ T cells and antigen presenting DCs. Nevertheless, deleting ICAM-1 from these DCs in our DC^ΔICAM-1^ mice did not impair monoclonal OVA specific CD8^+^ or CD4^+^ T cell proliferation or differentiation to PR8 encoded OVA peptides in MedLNs following influenza virus infection. Since cDCs express negligible levels of the low affinity LFA-1 ligand, ICAM-2, our results suggest that CD4^+^ and CD8^+^ T cells do not use their LFA-1 for priming and differentiation by migratory and resident DCs that present viral antigens to these T cells. Interestingly, on day 4 post infection with PR8 encoding the OT-I cognate ligand, SIINFEKL, OT-I CD8^+^ T cells in the MedLNs of ICAM-1/2^-/-^ mice proliferated and differentiated vigorously, whereas by day 7, their numbers declined in the ICAM-1/2^-/-^ MedLNs as well as in the ICAM-1/2^-/-^ lungs. Since a similar reduction of CD8^+^ effector T cells was not observed in the MedLNs of our conditional DC^ΔICAM-1^ mice (data not shown) these results suggest that newly generated virus specific CD8^+^ effector T cells use pools of stromal ICAM-1 and ICAM-2 (e.g., FRCs) for survival (90), a possibility that will need to be studied in future studies using new ICAM deficient mice models.

The MedLNs are major sites of B-cell responses following influenza virus infection (44). Both innate-like B-1 cells and conventional B-2 cells (44, 91) provide critical humoral protection against primary influenza virus infections. The polyclonal IgM produced by B-1 cells can initially bind a variety of different influenza strains (92), reduce viral load, present viral particles to incoming B-2 cells and help initiate the stronger T-dependent B-2 cell dependent anti-viral responses in extrafollicular regions prior to GC generation necessary for affinity maturation and generation of long lasting B cell memory (44).

In response to type I IFN produced in the infected respiratory tract, innate-like B-1 cells enter the MedLNs *via* HEVs (92). We found that this critical entry step was not inhibited in the ICAM-1/2^-/-^ MedLNs, arguing for usage of VLA-4 and possibly of Mac-1 expressed by these lymphocytes. The accumulation and generation of extrafollicular B-2 cells were also normal in ICAM-1/2^-/-^ MedLNs, reflecting a potential usage of α4 integrins also by these lymphocytes in the entry into ICAM-1 and ICAM-2 deficient MedLNs. Furthermore, B-2 lymphocytes entering ICAM-1/2^-/-^ LNs combated the primary influenza infection as efficiently as in WT MedLNs, suggesting normal interaction of these lymphocytes with FDCs, the key antigen presenting cells that comprise the B follicles. During early stages post infection, FDCs are involved in virus Ag uptake, early B-2 activation and subsequent B cell migration towards the T zone border of the follicles (93). Our findings suggest that in the ICAM-1/2^-/-^ MedLNs of influenza infected mice, B-2 cells collected normal activation and differentiation signals from FDCs, possibly *via* their VCAM-1 (94), which is likely sufficient to overcome the absence of ICAMs in the follicles. Later on, GCs develop from these follicles within 10-12 days post influenza infection (44) and persist up to several months after influenza infection clearance in both the MedLNs and in tertiary lymphoid organs such as nasal associated lymphoid tissues (NALTs) (95) and bronchial associated lymphoid tissues (BALTs) (96). The role of ICAMs in the persistence and functions of these distinct GCs remains to be explored.

Our unexpected results indicate that B-2 cells acquired normal T cell help in the ICAM-1/2^-/-^ MedLNs. Indeed upon encounter and activation by viral antigen-presenting DCs, viral-specific CD4^+^ T cells differentiated into early Tfh CD4^+^ cells which stably express CXCR5^+^, ICOS^+^, PD-1^+^, and are capable of migrating to the T/B border (97). The expression of the key Tfh transcription factor, BCL6, by these early Tfh cells was reduced in the absence of DC and FDC ICAMs, suggesting that their early maturation is partially ICAM-1 and ICAM-2 dependent. Nevertheless, these specialized B helper T effector cells gave rise to normal GC reactions on day 12 post infection. The strength of the TCR dependent immune synapses generated between monoclonal Ag presenting B cells and cognate Tfh cells was recently shown to be augmented by B cell expressed ICAM-1 and ICAM-2 (45). Our findings suggest, however, that in the course of influenza virus infection, ICAM-1 and ICAM-2 deficient polyclonal B cells can still efficiently present antigenic peptides to Tfh cells. Furthermore, high affinity antibodies (50), were normally generated in MedLNs deficient in both DC and B cell ICAMs. In addition, both virus specific memory CD4^+^ T cells and memory B cells, likely persisted in ICAM-1/2^-/-^ deficient MedLNs, since ICAM-1/2^-/-^ mice were fully protected from secondary infections with lethal doses of homosubtypic influenza and generated similar levels of virus specific IgGs during these recall responses.

In conclusion, our study suggests an exceptional flexibility in the usage of vascular, stromal, and epithelial integrin ligands by innate and adaptive leukocytes recruited to influenza infected lungs. Our study also highlights multiple compensatory roles of alternative integrin ligands expressed by lung vascular cells and by APCs operating in lung draining lymph nodes in the priming and differentiation of virus specific T and B lymphocytes elicited during primary influenza infection. Although initial homing of these lymphocytes into lung draining lymph nodes is defective, specific cDC subsets in ICAM-1/2^-/-^ mice appear hyperactivated and thereby compensate for the initially reduced accumulation of T and B cells in the lymph nodes of influenza-infected mice. The identification of these hyperactivated DC subsets in ICAM deficient lung draining lymph nodes eludes to DC suppressory mechanisms that depend on lung stromal ICAMs and affect cDC maturation and migration into lung draining lymph nodes during respiratory viral infections. New genetic models with multiple cell type specific deletions of ICAMs and other integrin ligands (e.g., VCAM-1) expressed by distinct stromal cells and cDC subsets could shed new light on where and how ICAMs and VCAM-1 regulate cDC maturation, lymphocyte differentiation, and lymphocyte memory generation in distinct viral infections. These new genetic models can also aid to new understanding of T cell education by ICAMs in the context of distinct lung tumors. These future studies may help in the design of improved vaccines against different pathogens and malignancies.

## Data availability statement

The original contributions presented in the study are included in the article/**Supplementary Materials**, further inquiries can be directed to the corresponding author.

## Ethics statement

The animal study was reviewed and approved by The Animal Care and Use Committee of the Weizmann Institute of Science.

## Author contributions

SK designed and performed a major part of the *in vivo* experiments, analyzed the data, and contributed to the writing of the manuscript. OR, AS, MK and EP-K assisted with *in vivo* experiments. HA and NE produced purified PR8 for ELISA determinations, quantified the pulmonary PR8 viral load *via* qPRC, conducted the ELISA for IgG antibody detection against PR8 and assisted with the detection of IgG2a. JE performed IF staining of MedLNs. OR and MK assisted with LSM imaging and data analysis. YA assisted with data acquisition of LSM. FC imaged neutrophils inside lung capillaries by pulmonary intravital imaging. PK supervised the pulmonary intravital imaging experiments. NG provided the various PR8 variants and advised on several experimental directions. RA supervised all the experiments and wrote the manuscript. All authors contributed to the article and approved the submitted version.

## References

[1] Holt PG Strickland DH Wikström ME Jahnsen FL. Regulation of immunological homeostasis in the respiratory tract. Nat Rev Immunol (2008) 8:142–52. doi: 10.1038/nri2236 18204469

[2] Alon R Sportiello M Kozlovski S Kumar A Reilly EC Zarbock A. Leukocyte trafficking to the lungs and beyond: Lessons from influenza for COVID-19. Nat Rev Immunol (2021) 21:49–64. doi: 10.1038/s41577-020-00470-2 33214719 PMC7675406

[3] Denney L Ho LP. The role of respiratory epithelium in host defence against influenza virus infection. BioMed J (2018) 41:218–33. doi: 10.1016/J.BJ.2018.08.004 PMC6197993 30348265

[4] Ley K Reutershan J. Leucocyte-endothelial interactions in health and disease. Handb Exp Pharmacol (2006) 176(Pt 2):97–133. doi: 10.1007/3-540-36028-X_4 16999226

[5] Borja-Cacho D Matthews J. Blockade of leucocyte function-associated antigen-1 (LFA-1) decreases lymphocyte trapping in the normal pulmonary vasculature: Studies in the isolated buffer-perfused rat lung. Nano (2008) 6:2166–71. doi: 10.1021/nl061786n.Core-Shell PMC1905710 10931156

[6] Worbs T Hammerschmidt SI Förster R. Dendritic cell migration in health and disease. Nat Rev Immunol (2017) 17:30–48. doi: 10.1038/nri.2016.116 27890914

[7] Allingham MJ van Buul JD Burridge K. ICAM-1-mediated, src- and Pyk2-dependent vascular endothelial cadherin tyrosine phosphorylation is required for leukocyte transendothelial migration. J Immunol (2007) 179:4053–64. doi: 10.4049/jimmunol.179.6.4053 17785844

[8] Cornelius LA Taylor JT Degitz K Li LJ Lawley TJ Caughman SW. A 5’ portion of the ICAM-1 gene confers tissue-specific differential expression levels and cytokine responsiveness. J Invest Dermatol (1993) 100:753–8. doi: 10.1111/1523-1747.ep12476300 8098727

[9] Ray SJ Franki SN Pierce RH Dimitrova S Koteliansky V Sprague AG. The collagen binding alpha1beta1 integrin VLA-1 regulates CD8 T cell-mediated immune protection against heterologous influenza infection. Immunity (2004) 20:167–79. doi: 10.1016/S1074-7613(04)00021-4 14975239

[10] Sundd P Gutierrez E Koltsova EK Kuwano Y Fukuda S Pospieszalska MK. “Slings” enable neutrophil rolling at high shear. Nature (2012) 488:399–403. doi: 10.1038/nature11248nature11248 22763437 PMC3433404

[11] Constantin G Majeed M Giagulli C Piccio L Kim JY Butcher EC. Chemokines trigger immediate β2 integrin affinity and mobility changes: Differential regulation and roles in lymphocyte arrest under flow. Immunity (2000) 13:759–69. doi: 10.1016/S1074-7613(00)00074-1 11163192

[12] Hubbard AK Rothlein R. Intercellular adhesion molecule-1 (ICAM-1) expression and cell signaling cascades. Free Radic Biol Med (2000) 28(9):1379–86. doi: 10.1016/S0891-5849(00)00223-9 10924857

[13] Dustin ML. The immunological synapse. Cancer Immunol Res (2014) 2:1023. doi: 10.1158/2326-6066.CIR-14-0161 25367977 PMC4692051

[14] Caton ML Smith-Raska MR Reizis B. Notch-RBP-J signaling controls the homeostasis of CD8- dendritic cells in the spleen. J Exp Med (2007) 204:1653–64. doi: 10.1084/jem.20062648 PMC2118632 17591855

[15] Feigelson SW Solomon A Biram A Hatzav M Lichtenstein M Regev O. ICAMs are not obligatory for functional immune synapses between naive CD4 T cells and lymph node DCs. Cell Rep (2018) 22:849–59. doi: 10.1016/j.celrep.2017.12.103 29420172

[16] Looney MR Thornton EE Sen D Lamm WJ Glenny RW Krummel MF. Stabilized imaging of immune surveillance in the mouse lung. Nat Methods (2011) 8:91–6. doi: 10.1038/nmeth.1543 PMC3076005 21151136

[17] Yipp BG Kim JH Lima R Zbytnuik LD Petri B Swanlund N. The lung is a host defense niche for immediate neutrophil-mediated vascular protection. Sci Immunol (2017) 2. doi: 10.1126/sciimmunol.aam8929 PMC5472445 28626833

[18] Regev O Kizner M Roncato F Dadiani M Saini M Castro-Giner F. ICAM-1 on breast cancer cells suppresses lung metastasis but is dispensable for tumor growth and killing by cytotoxic T cells. Front Immunol (2022) 13:849701. doi: 10.3389/fimmu.2022.849701 35911772 PMC9328178

[19] Quah BJ Warren HS Parish CR. Monitoring lymphocyte proliferation *in vitro* and *in vivo* with the intracellular fluorescent dye carboxyfluorescein diacetate succinimidyl ester. Nat Protoc (2007) 2:2049–56. doi: 10.1038/nprot.2007.296 17853860

[20] Thomas G Tacke R Hedrick CC Hanna RN. Nonclassical patrolling monocyte function in the vasculature. Arterioscler Thromb Vasc Biol (2015) 35(6):1306–16. doi: 10.1161/ATVBAHA.114.304650 PMC4441550 25838429

[21] Doerschuk CM. Leukocyte trafficking in alveoli and airway passages. Respir Res (2000) 1:136–40. doi: 10.1186/rr24 PMC59559 11667977

[22] Petrovich E Feigelson SW Stoler-Barak L Hatzav M Solomon A Bar-Shai A. Lung ICAM-1 and ICAM-2 support spontaneous intravascular effector lymphocyte entrapment but are not required for neutrophil entrapment or emigration inside endotoxin-inflamed lungs. FASEB J (2016) 30(5):1767–78. doi: 10.1096/fj.201500046 26823454

[23] Auffray C Fogg D Garfa M Elain G Join-Lambert O Kayal S. Monitoring of blood vessels and tissues by a population of monocytes with patrolling behavior. Science (2007) 317:666–70. doi: 10.1126/science.1142883 17673663

[24] Chen J Ganguly A Mucsi AD Meng J Yan J Detampel P. Strong adhesion by regulatory T cells induces dendritic cell cytoskeletal polarization and contact-dependent lethargy. J Exp Med (2017) 214:327–38. doi: 10.1084/jem.20160620 PMC5294852 28082358

[25] Gillich A Zhang F Farmer CG Travaglini KJ Tan SY Gu M. Capillary cell-type specialization in the alveolus. Nat (2020) 586(7831):785–9. doi: 10.1038/s41586-020-2822-7 PMC7721049 33057196

[26] Topham DJ Tripp RA Doherty PC. CD8+ T cells clear influenza virus by perforin or fas-dependent processes. J Immunol (1997) 159(11):5197–200. doi: 10.4049/jimmunol.159.11.5197 9548456

[27] Bouvier NM Lowen AC. Animal models for influenza virus pathogenesis and transmission. Viruses (2010) 2(8):1530–63. doi: 10.3390/v20801530 PMC3063653 21442033

[28] Othumpangat S Noti JD Mcmillen CM Beezhold DH. ICAM-1 regulates the survival of influenza virus in lung epithelial cells during the early stages of infection. Virology (2016) 487:85–94. doi: 10.1016/j.virol.2015.10.005 PMC4719144 26499045

[29] Nguyen HH Zemlin M Ivanov II Andrasi J Zemlin C Vu HL. Heterosubtypic immunity to influenza a virus infection requires a properly diversified antibody repertoire. J Virol (2007) 81:9331–8. doi: 10.1128/jvi.00751-07 PMC1951409 17567700

[30] Rangel-moreno J Carragher DM Misra RS Kusser K Hartson L Moquin A. B cells promote resistance to heterosubtypic strains of influenza via multiple mechanisms. J Immunol. (2009) 180:454–63. doi: 10.4049/jimmunol.180.1.454 PMC2712821 18097047

[31] Zhong W Reinherz EL. *In vivo* selection of a TCR vβ repertoire directed against an immunodominant influenza virus CTL epitope. Int Immunol (2004) 16:1549–59. doi: 10.1093/intimm/dxh156 15351787

[32] Carlin LE Hemann EA Zacharias ZR Heusel JW Legge KL. Natural killer cell recruitment to the lung during influenza a virus infection is dependent on CXCR3, CCR5, and virus exposure dose. Front Immunol (2018) 9:781. doi: 10.3389/fimmu.2018.00781 29719539 PMC5913326

[33] Tate MD Ioannidis LJ Croker B Brown LE Brooks AG Reading PC. The role of neutrophils during mild and severe influenza virus infections of mice. PloS One (2011) 6:e17618. doi: 10.1371/journal.pone.0017618 21423798 PMC3056712

[34] Zheng J Perlman S. Immune responses in influenza a virus and human coronavirus infections: An ongoing battle between the virus and host. Curr Opin Virol (2018) 28:43. doi: 10.1016/J.COVIRO.2017.11.002 29172107 PMC5835172

[35] Galani IE Andreakos E. Neutrophils in viral infections: Current concepts and caveats. J Leukoc Biol (2015) 98:557–64. doi: 10.1189/JLB.4VMR1114-555R 26160849

[36] van der Sandt CE Kreijtz JHCM Rimmelzwaan GF. Evasion of influenza a viruses from innate and adaptive immune responses. Viruses (2012) 4:1438–76. doi: 10.3390/V4091438 PMC3499814 23170167

[37] Lim YC Wakelin MW Henault L Goetz DJ Yednock T Cabanas C. Alpha4beta1-integrin activation is necessary for high-efficiency T-cell subset interactions with VCAM-1 under flow. Microcirculation (2000) 7:201–14. doi: 10.1111/j.1549-8719.2000.tb00121.x 10901499

[38] Connor EM Eppihimer MJ Morise Z Granger DN Grisham MB. Expression of mucosal addressin cell adhesion molecule-1 (MAdCAM-1) in acute and chronic inflammation. J Leukoc Biol (1999) 65:349–55. doi: 10.1002/jlb.65.3.349 10080539

[39] Caucheteux SM Torabi-Parizi P Paul WE. Analysis of naive lung CD4 T cells provides evidence of functional lung to lymph node migration. Proc Natl Acad Sci (2013) 110:1821–6. doi: 10.1073/pnas.1221306110 PMC3562823 23319636

[40] Renegar KB Small PA Boykins LG Wright PF. Role of IgA versus IgG in the control of influenza viral infection in the murine respiratory tract. J Immunol (2004) 173:1978–86. doi: 10.4049/JIMMUNOL.173.3.1978 15265932

[41] GeurtsvanKessel CH Lambrecht BN. Division of labor between dendritic cell subsets of the lung. Mucosal Immunol (2008) 1:442–50. doi: 10.1038/mi.2008.39 19079211

[42] Padilla-Quirarte HO Lopez-Guerrero DV Gutierrez-Xicotencatl L Esquivel-Guadarrama F. Protective antibodies against influenza proteins. Front Immunol (2019) 10:1677. doi: 10.3389/fimmu.2019.01677 31379866 PMC6657620

[43] Choi YS Baumgarth N. Dual role for b-1a cells in immunity to influenza virus infection. J Exp Med (2008) 205:3053–64. doi: 10.1084/jem.20080979 PMC2605232 19075288

[44] Baumgarth N. How specific is too specific? b-cell responses to viral infections reveal the importance of breadth over depth. Immunol Rev (2013) 255:82–94. doi: 10.1111/imr.12094 23947349 PMC3748619

[45] Zaretsky I Atrakchi O Mazor RD Stoler-Barak L Biram A Feigelson SW. ICAMs support b cell interactions with T follicular helper cells and promote clonal selection. J Exp Med (2017) 214:3435–48. doi: 10.1084/jem.20171129 PMC5679169 28939548

[46] Ballesteros-Tato A León B Lund FE Randall TD. Temporal changes in dendritic cell subsets, cross-priming and costimulation *via* CD70 control CD8+ T cell responses to influenza. Nat Immunol (2010) 11:216–24. doi: 10.1038/ni.1838 PMC2822886 20098442

[47] McGill J Van Rooijen N Legge KL. Protective influenza-specific CD8 T cell responses require interactions with dendritic cells in the lungs. J Exp Med (2008) 205:1635–46. doi: 10.1084/jem.20080314 PMC2442641 18591411

[48] Román E Miller E Harmsen A Wiley J von Andrian UH Huston G. CD4 effector T cell subsets in the response to influenza. J Exp Med (2002) 196:957–68. doi: 10.1084/jem.20021052 PMC2194021 12370257

[49] Brewitz A Eickhoff S Dähling S Quast T Bedoui S Kroczek RA. CD8 + T cells orchestrate pDC-XCR1 + dendritic cell spatial and functional cooperativity to optimize priming. Immunity (2017) 46:205–19. doi: 10.1016/j.immuni.2017.01.003 PMC5362251 28190711

[50] Hocart MJ Mackenzie JS Stewart GA. The immunoglobulin G subclass responses of mice to influenza a virus: The effect of mouse strain, and the neutralizing abilities of individual protein a-purified subclass antibodies. J Gen Virol (1989) 70(Pt 9):2439–48. doi: 10.1099/0022-1317-70-9-2439 2778440

[51] Lukacher AE Braciale VL Braciale TJ. *In vivo* effector function of influenza virus-specific cytotoxic T lymphocyte clones is highly specific. J Exp Med (1984) 160:814–26. doi: 10.1084/jem.160.3.814 PMC2187390 6206190

[52] La Gruta NL Turner SJ. T Cell mediated immunity to influenza: Mechanisms of viral control. Trends Immunol (2014) 35:396–402. doi: 10.1016/J.IT.2014.06.004 25043801

[53] Cook-Mills JM Marchese ME Abdala-Valencia H. Vascular cell adhesion molecule-1 expression and signaling during disease: Regulation by reactive oxygen species and antioxidants. Antioxidants Redox Signal (2011) 15:1607–38. doi: 10.1089/ars.2010.3522 PMC3151426 21050132

[54] Nakanishi Y Lu B Gerard C Iwasaki A. CD8(+) T lymphocyte mobilization to virus-infected tissue requires CD4(+) T-cell help. Nature (2009) 462:510–3. doi: 10.1038/nature08511 PMC2789415 19898495

[55] La Gruta NL Kedzierska K Stambas J Doherty PC. A question of self-preservation: Immunopathology in influenza virus infection. Immunol Cell Biol (2007) 85:85–92. doi: 10.1038/sj.icb.7100026 17213831

[56] Deckhut AM Allan W McMickle A Eichelberger M Blackman MA Doherty PC. Prominent usage of V beta 8.3 T cells in the h-2Db-restricted response to an influenza a virus nucleoprotein epitope. J Immunol (1993) 151:2658–26566. doi: 10.4049/jimmunol.151.5.2658 7689611

[57] Takamura S. Niches for the long-term maintenance of tissue-resident memory T cells. Front Immunol (2018) 9:1214. doi: 10.3389/fimmu.2018.01214 29904388 PMC5990602

[58] Ray SJ Franki SN Pierce RH Dimitrova S Koteliansky V Sprague AG. The collagen binding α1β1 integrin VLA-1 regulates CD8 T cell-mediated immune protection against heterologous influenza infection. Immunity (2004) 20:167–79. doi: 10.1016/S1074-7613(04)00021-4 14975239

[59] Topham DJ Reilly EC Emo KL Sportiello M. Formation and maintenance of tissue resident memory CD8+ T cells after viral infection. Pathogens (2019) 8:1–9. doi: 10.3390/pathogens8040196 PMC6963622 31635290

[60] Zens KD Chen JK Farber DL. Vaccine-generated lung tissue–resident memory T cells provide heterosubtypic protection to influenza infection. JCI Insight (2019) 1:1–12. doi: 10.1172/jci.insight.85832 PMC4959801 27468427

[61] Duan S Thomas PG. Balancing immune protection and immune pathology by CD8+ T-cell responses to influenza infection. Front Immunol (2016) 7:25. doi: 10.3389/fimmu.2016.00025 26904022 PMC4742794

[62] Wein AN McMaster SR Takamura S Dunbar PR Cartwright EK Hayward SL. CXCR6 regulates localization of tissue-resident memory CD8 T cells to the airways. J Exp Med (2019) 216:2748–62. doi: 10.1084/jem.20181308 PMC6888981 31558615

[63] Teijaro JR Turner D Pham Q Wherry EJ Lefrançois L Farber DL. Cutting edge: Tissue-retentive lung memory CD4 T cells mediate optimal protection to respiratory virus infection. J Immunol (2011) 187:5510–4. doi: 10.4049/JIMMUNOL.1102243 PMC3221837 22058417

[64] Kim TS Sun J Braciale TJ. T Cell responses during influenza infection: Getting and keeping control. Trends Immunol (2011) 32:225–31. doi: 10.1016/j.it.2011.02.006 PMC3090469 21435950

[65] Weis W Brown JH Cusack S Paulson JC Skehel JJ Wiley DC. Structure of the influenza virus haemagglutinin complexed with its receptor, sialic acid. Nat (1988) 333(6172):426–31. doi: 10.1038/333426a0 3374584

[66] Kumlin U Olofsson S Dimock K Arnberg N. Sialic acid tissue distribution and influenza virus tropism. Influenza Other Respi Viruses (2008) 2:147. doi: 10.1111/J.1750-2659.2008.00051.X PMC4941897 19453419

[67] Hufford MM Richardson G Zhou H Manicassamy B García-Sastre A Enelow RI. Influenza-infected neutrophils within the infected lungs act as antigen presenting cells for anti-viral CD8+ T cells. PloS One (2012) 7:e46581. doi: 10.1371/journal.pone.0046581 23056353 PMC3466305

[68] Hamada H Bassity E Flies A Strutt TM Garcia-Hernandez M de L McKinstry KK. Multiple redundant effector mechanisms of CD8+ T cells protect against influenza infection. J Immunol (2013) 190:296–306. doi: 10.4049/JIMMUNOL.1200571 23197262 PMC3864858

[69] Herold S Becker C Ridge KM Budinger GRS. Influenza virus-induced lung injury: pathogenesis and implications for treatment. Eur Respir J (2015) 45:1463–78. doi: 10.1183/09031936.00186214 25792631

[70] Schneberger D Sethi RS Singh B. Comparative view of lung vascular endothelium of cattle, horses, and water buffalo. Adv Anat Embryology Cell Biol (2018) 228:21–39. doi: 10.1007/978-3-319-68483-3_2 29288384

[71] Galkina E Thatte J Dabak V Williams MB Ley K Braciale TJ. Preferential migration of effector CD8+T cells into the interstitium of the normal lung. J Clin Invest (2005) 115:3473–83. doi: 10.1172/JCI24482 PMC1288831 16308575

[72] Wanner A. Circulation of the airway mucosa. J Appl Physiol (1989) 67:917–25. doi: 10.1152/jappl.1989.67.3.917 2571606

[73] Topham DJ Reilly EC. Tissue-resident memory CD8+ T cells: From phenotype to function. Front Immunol (2018) 9:515. doi: 10.3389/fimmu.2018.00515 29632527 PMC5879098

[74] Schmidt EP Yang Y Janssen WJ Gandjeva A Perez MJ Barthel L. The pulmonary endothelial glycocalyx regulates neutrophil adhesion and lung injury during experimental sepsis. Nat Med (2012) 18:1217–23. doi: 10.1038/nm.2843 PMC3723751 22820644

[75] Schittenhelm L Hilkens CM Morrison VL. β2 integrins as regulators of dendritic cell, monocyte, and macrophage function. Front Immunol (2017) 8:1866/BIBTEX. doi: 10.3389/FIMMU.2017.01866/BIBTEX 29326724 PMC5742326

[76] Tak T Rygiel TP Karnam G Bastian OW Boon L Viveen M. Neutrophil-mediated suppression of influenza-induced pathology requires CD11b/CD18 (MAC-1). Am J Respir Cell Mol Biol (2018) 58:492–9. doi: 10.1165/rcmb.2017-0021OC 29141155

[77] Girard JP Moussion C Forster R. HEVs, lymphatics and homeostatic immune cell trafficking in lymph nodes. Nat Rev Immunol (2012) 12:762–73. doi: 10.1038/nri3298 23018291

[78] Foo SY Phipps S. Regulation of inducible BALT formation and contribution to immunity and pathology. Mucosal Immunol (2010) 3:537–44. doi: 10.1038/mi.2010.52 20811344

[79] Bogoslowski A Wijeyesinghe S Lee W-Y Chen C-S Alanani S Jenne C. Neutrophils recirculate through lymph nodes to survey tissues for pathogens. J Immunol (2020) 204(9):2552–61. doi: 10.4049/jimmunol.2000022 32205425

[80] Ho AWS Prabhu N Betts RJ Ge MQ Dai X Hutchinson PE. Lung CD103 + dendritic cells efficiently transport influenza virus to the lymph node and load viral antigen onto MHC class I for presentation to CD8 T cells. J Immunol (2011) 187:6011–21. doi: 10.4049/jimmunol.1100987 22043017

[81] Topham DJ Tripp RA Hamilton-Easton AM Sarawar SR Doherty PC. Quantitative analysis of the influenza virus-specific CD4+ T cell memory in the absence of b cells and ig. J Immunol (1996) 157:2947–52. 8816401

[82] Boscacci RT Pfeiffer F Gollmer K Sevilla AI Martin AM Soriano SF. Comprehensive analysis of lymph node stroma-expressed ig superfamily members reveals redundant and nonredundant roles for ICAM-1, ICAM-2, and VCAM-1 in lymphocyte homing. Blood (2010) 116:915–25. doi: 10.1182/blood-2009-11-254334 PMC3324225 20395417

[83] Guilliams M Lambrecht BN Hammad H. Division of labor between lung dendritic cells and macrophages in the defense against pulmonary infections. Mucosal Immunol (2013) 6:464–73. doi: 10.1038/mi.2013.14 23549447

[84] Lambrecht BN Hammad H. Lung dendritic cells in respiratory viral infection and asthma: From protection to immunopathology. Annu Rev Immunol (2012) 30:243–70. doi: 10.1146/annurev-immunol-020711-075021 22224777

[85] Hemann EA Sjaastad LE Langlois RA Legge KL. Plasmacytoid dendritic cells require direct infection to sustain the pulmonary influenza a virus-specific CD8 T cell response. J Virol (2016) 90:2830–7. doi: 10.1128/jvi.02546-15 PMC4810669 26719269

[86] Kim TS Braciale TJ. Respiratory dendritic cell subsets differ in their capacity to support the induction of virus-specific cytotoxic CD8+ T cell responses. PloS One (2009) 4:e4204. doi: 10.1371/journal.pone.0004204 19145246 PMC2615220

[87] Dudziak D Kamphorst AO Heidkamp GF Buchholz VR Trumpfheller C Yamazaki S. Differential antigen processing by dendritic cell subsets *in vivo* . Science (2007) 315:107–11. doi: 10.1126/science.1136080 17204652

[88] Ng SL Teo YJ Setiagani YA Karjalainen K Ruedl C. Type 1 conventional CD103+ dendritic cells control effector CD8+ T cell migration, survival, and memory responses during influenza infection. Front Immunol (2018) 9:3043. doi: 10.3389/fimmu.2018.03043 30622538 PMC6308161

[89] Bosteels C Neyt K Vanheerswynghels M van Helden MJ Sichien D Debeuf N. Inflammatory type 2 cDCs acquire features of cDC1s and macrophages to orchestrate immunity to respiratory virus infection. Immunity (2020) 52(6):1039–1056.e9. doi: 10.1016/j.immuni.2020.04.005 PMC7207120 32392463

[90] Link A Vogt TK Favre S Britschgi MR Acha-Orbea H Hinz B. Fibroblastic reticular cells in lymph nodes regulate the homeostasis of naive T cells. Nat Immunol (2007) 8:1255–65. doi: 10.1038/ni1513 17893676

[91] Lam JH Baumgarth N. The multifaceted b cell response to influenza virus. J Immunol (2019) 202:351–9. doi: 10.4049/jimmunol.1801208 PMC6327962 30617116

[92] Baumgarth N Herman OC Jager GC Brown L Herzenberg LA Herzenberg LA. Innate and acquired humoral immunities to influenza virus are mediated by distinct arms of the immune system. Proc Natl Acad Sci U.S.A. (1999) 96:2250–5. doi: 10.1073/pnas.96.5.2250 PMC26769 10051627

[93] Okada T Miller MJ Parker I Krummel MF Neighbors M Hartley SB. Antigen-engaged b cells undergo chemotaxis toward the T zone and form motile conjugates with helper T cells. PloS Biol (2005) 3:e150. doi: 10.1371/journal.pbio.0030150 15857154 PMC1088276

[94] Bajenoff M Egen JG Koo LY Laugier JPP Brau F Glaichenhaus N. Stromal cell networks regulate lymphocyte entry, migration, and territoriality in lymph nodes. Immunity (2006) 25:989–1001. doi: 10.1016/j.immuni.2006.10.011 17112751 PMC2692293

[95] Kiyono H Fukuyama S. NALT- versus peyer’s-patch-mediated mucosal immunity. Nat Rev Immunol (2004) 4:699–710. doi: 10.1038/NRI1439 15343369 PMC7097243

[96] Geurtsvankessel CH Willart MAM Bergen IM Van Rijt LS Muskens F Elewaut D. Dendritic cells are crucial for maintenance of tertiary lymphoid structures in the lung of influenza virus-infected mice. J Exp Med (2009) 206:2339–49. doi: 10.1084/jem.20090410 PMC2768850 19808255

[97] Chen M Guo Z Ju W Ryffel B He X Zheng SG. The development and function of follicular helper T cells in immune responses. Cell Mol Immunol (2012) 9:375–9. doi: 10.1038/cmi.2012.18 PMC4000446 22659733

