# Supplementary material for: ICAMs are dispensable for influenza clearance and anti-viral humoral and cellular immunity
Source: Front Immunol. 2023 Feb 21;13:1041552. doi: 10.3389/fimmu.2022.1041552 (PMC9988921; doi:10.3389/fimmu.2022.1041552)
Supplement: Supplementary file 1 [file DataSheet_1.pdf]

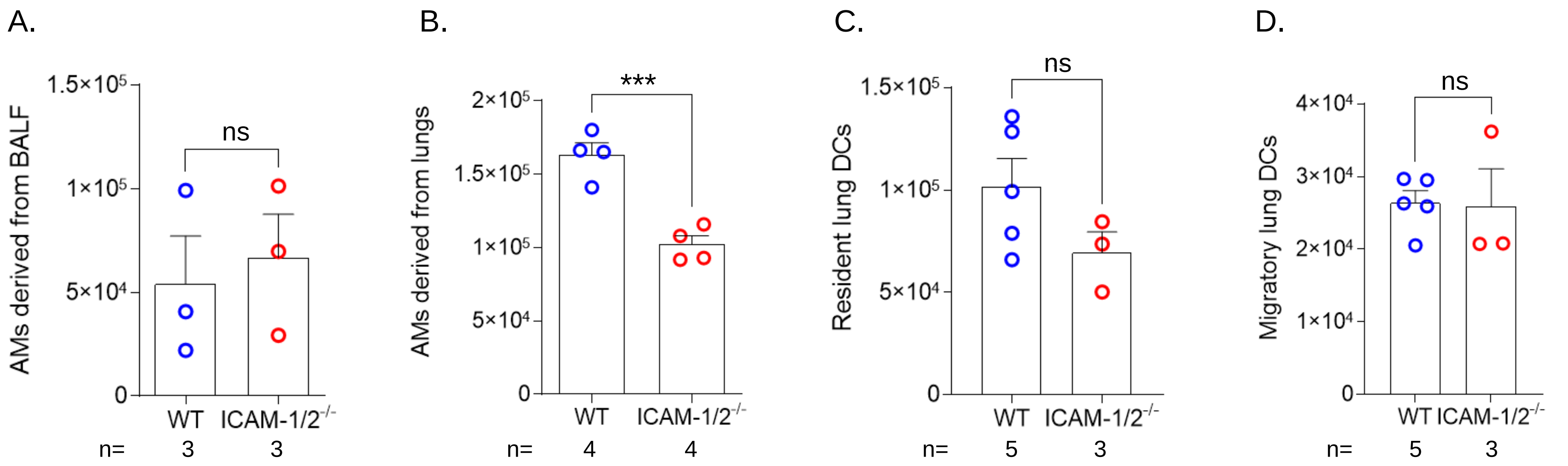

Supplementary Figure 1

The steady state cellularity of AMs and DCs in WT and ICAM-1/2$^{-/-}$ lungs. AMs (SiglecF^{+}CD11c^{+}) derived from (A) BALF or from (B) total cell suspension of lungs subjected to lavage were analyzed by flow cytometry. The numbers of (C) resident (MHCII^{int}CD11c^{high}) and (D) migratory (MHCII^{hi}CD11c^{int}) DCs recovered from WT (blue) and ICAM1/2$^{-/-}$ (red) lungs at steady state were determined by flow cytometry. Statistical significance was determined by two-tailed, unpaired Student's t tests. ***$P < 0.001$, ns, not significant. The numbers of each experimental group are indicated in the graphs. The error bars indicate the SEM of each measurement.

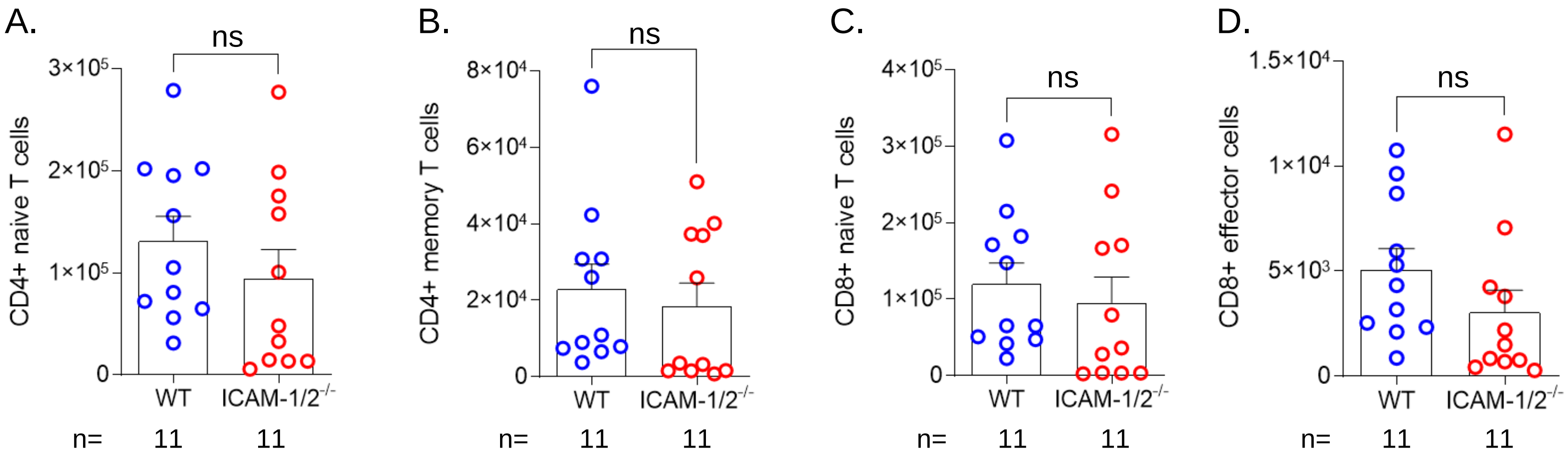

Supplementary Figure 2

Naïve and effector memory CD4^{+} and CD8^{+} T cells are retained in the lungs independently of ICAMs. (A) Naïve and (B) effector memory CD4^{+} T cells and (C) naïve and (D) effector memory CD8^{+} T cells were recovered from the lungs of either WT (blue) or ICAM-1/2$^{-/-}$ (red) mice and analyzed by flow cytometry. Naïve; CD44^{-}CD62L^{+}; Effector memory CD44^{+}CD62L^{-}. Statistical significance was determined by two-tailed, unpaired Student's t tests. ns, not significant. The numbers of each experimental group are indicated in the graphs. The error bars indicate the SEM of each measurement.

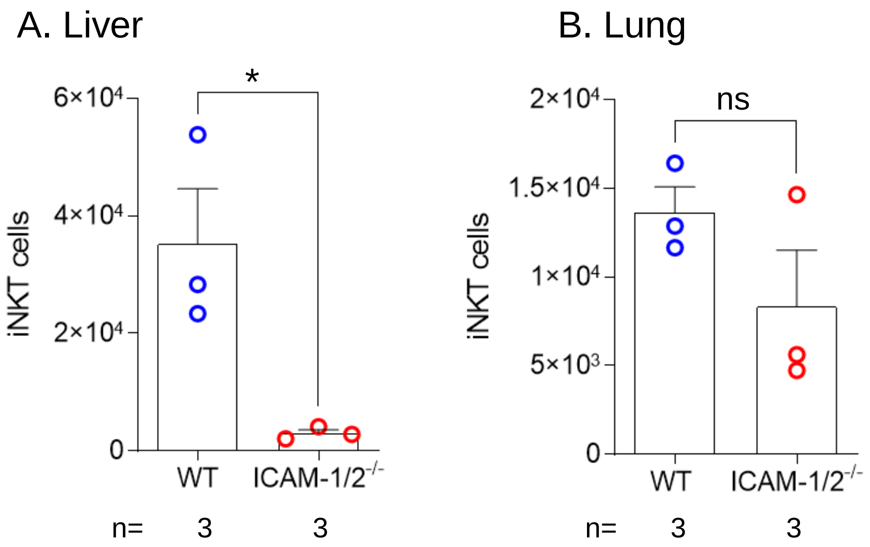

Supplementary Figure 3

iNKT cellularity in the liver and lung of WT and ICAM-1/2$^{-/-}$ mice at steady state. (A) Liver and (B) lung iNKT cells of either WT (blue) or ICAM-1/2$^{-/-}$ (red) mice were determined by CD45^{+}CD11b^{+}CD3^{+}PBS-57-loaded tetramer^{+} using: anti-CD45 mAb (Biolegend, Cat. 103108, clone 30-F11), anti-CD11b mAb(Biolegend, Cat. 101228, clone M1/70), anti-CD3 mAb (Biolegend, Cat. 100312, clone 145-2c11) and the PE-conjugated PBS-57-loaded or unloaded CD1d tetramer (NIH, NIAID tetramer core facility, Atlanta GA). Statistical significance was determined by two-tailed, unpaired Student’s t tests. *$P < 0.05$, ns, not significant. The numbers of each experimental group are indicated in the graphs. The error bars indicate the SEM of each measurement.

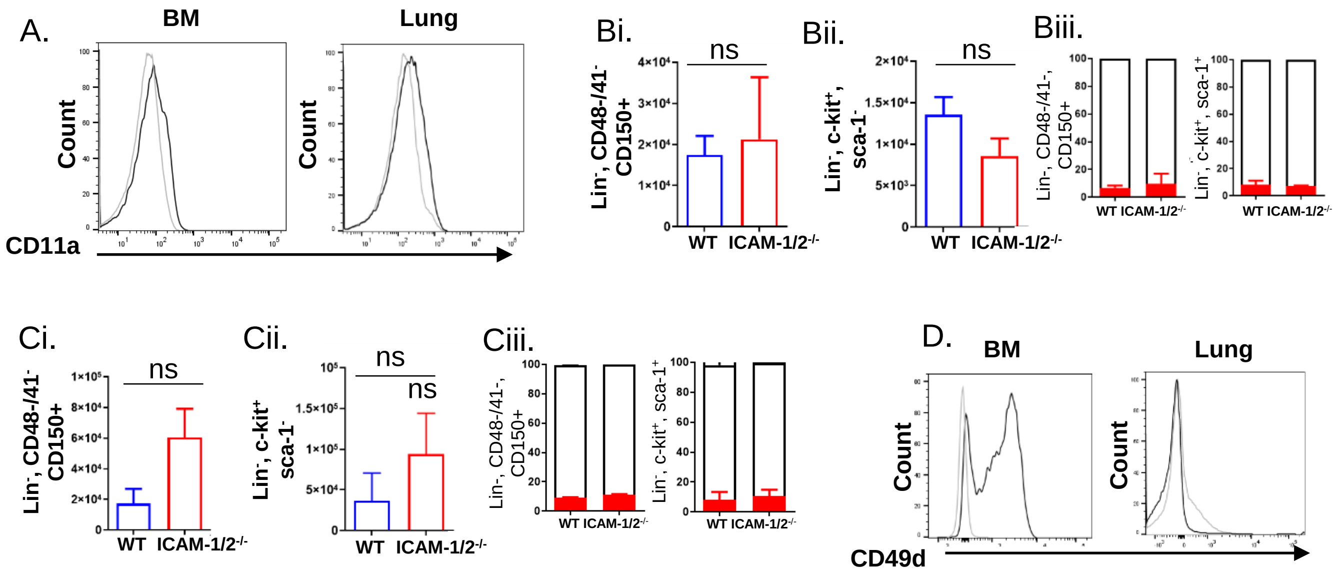

Supplementary Figure 4

Cellularity of HSCs recovered in the bone marrow and lungs of WT and ICAM-1/2$^{-/-}$ mice. (A) Flow cytometry analysis of CD11a surface expression (black) on HSCs (Lin^{-}CD48-/41^{-}CD150^{+}) in the bone marrow (BM) and lungs of WT mice. Isotype control staining is shown in grey. (Bi,Bii) The numbers of the Lin^{-}CD48-/41^{-}CD150^{+} and of the Lin^{-}kit^{+}sca-1^{+} BM derived HSC subsets isolated from WT (blue) and ICAM-1/2$^{-/-}$ (red) mice and their partition between vascular and extravascular compartments (Biii). (Ci,Cii) The numbers of distinct lung derived HSC subsets isolated from WT and ICAM-1/2$^{-/-}$ mice and their partition between vascular and extravascular compartments (Ciii). The markers of each subset are indicated near the Y axis. (D) Flow cytometry analysis of α4 (cd49D) integrin surface expression on Lin^{-}CD48-/41^{-}CD150^{+} HSCs (black). Isotype control staining is shown in grey. Statistical significance was determined by two-tailed, unpaired Student’s t tests. ns, not significant. n=3 for each group. The error bars indicate the SEM of each measurement.

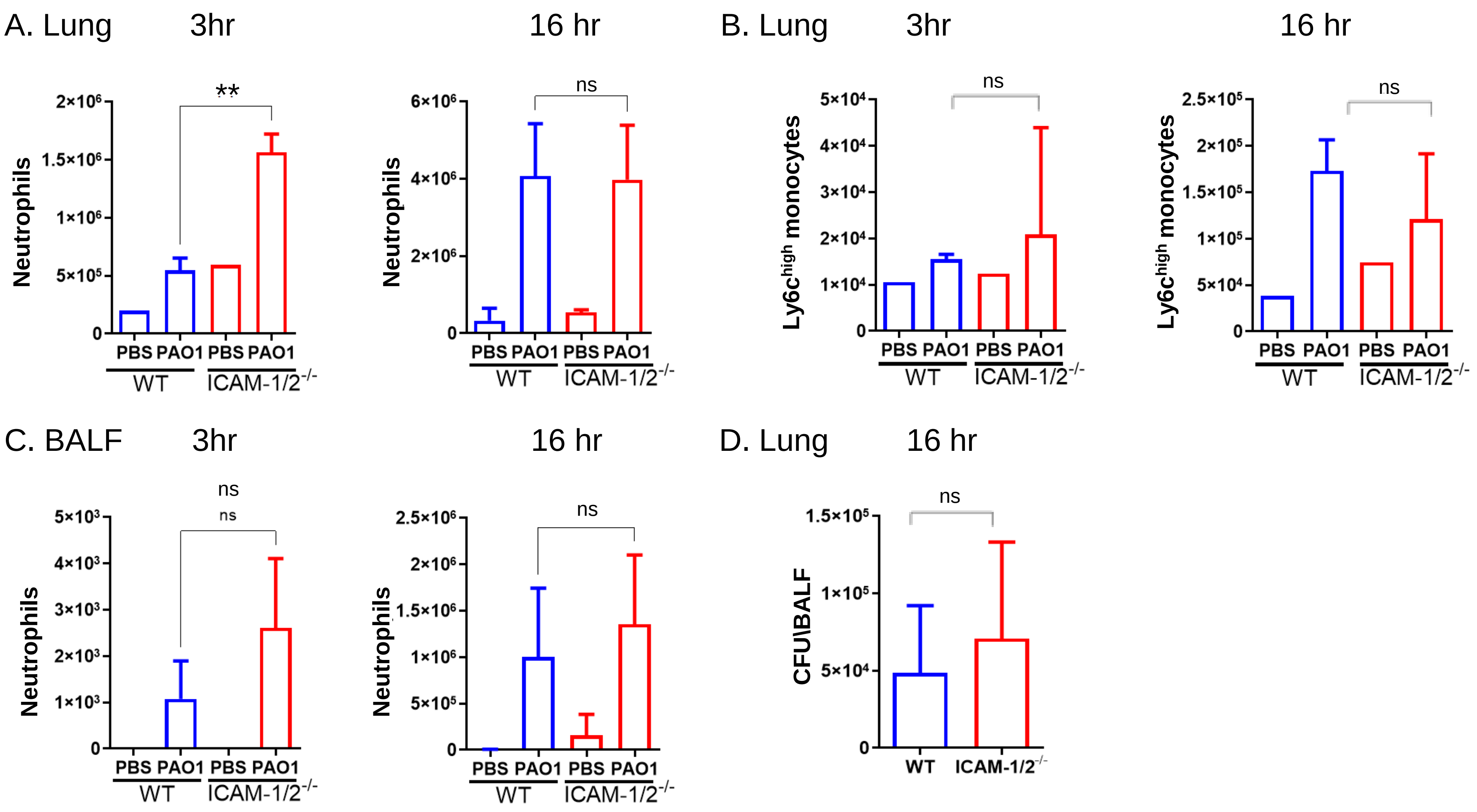

Supplementary Figure 5

Recruitment of neutrophils and Ly6C^{hi} monocytes to lungs of WT and ICAM-1/2$^{-/-}$ mice following Pseudomonas aeruginosa infection.
WT (blue) or ICAM-1/2$^{-/-}$ (red) mice were I.T. infected with 3x10^{6} CFU of the PAO1 strain of the gram-negative bacteria Pseudomonas aeruginosa. (A) Kinetics of neutrophil accumulation in infected lungs. (B) Kinetics of Ly6C^{hi} monocyte accumulation in infected lungs. (C) Kinetics of neutrophil entry to the bronchoalveolar space determined in BALF 3 hr and 16 hr post infection. (D) The levels of PAO1 detected in the BALF of the compared mice groups 16 hr post infection. Lungs were completely cleared of PAO1 by 48 hr post infection (data not shown). Statistical significance was determined by two-tailed, unpaired Student's t tests. **$P < 0.01$, ns, not significant. n=2-8 for each group. The error bars indicate the SEM of each measurement.

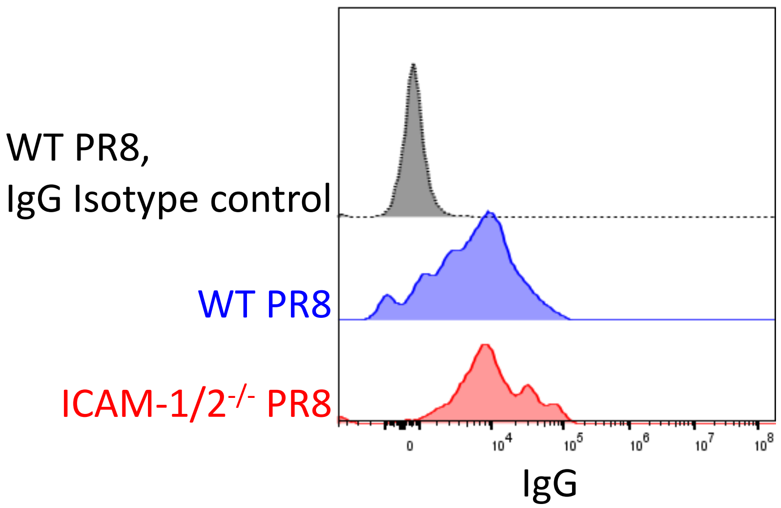

Supplementary Figure 6

Flow cytometry expression levels of surface IgG on extrafollicular B cells in the MedLN of infected WT and ICAM-1/2 $^{-/-}$ mice.

The indicated mice groups were infected I.N. with a sublethal dose of PR8-OVAII and on day 5 their MedLNs were isolated. Histograms depict IgG surface expression on extrafollicular B cells ($CD138^{+}B220^{-}$) recovered from the MedLNs.

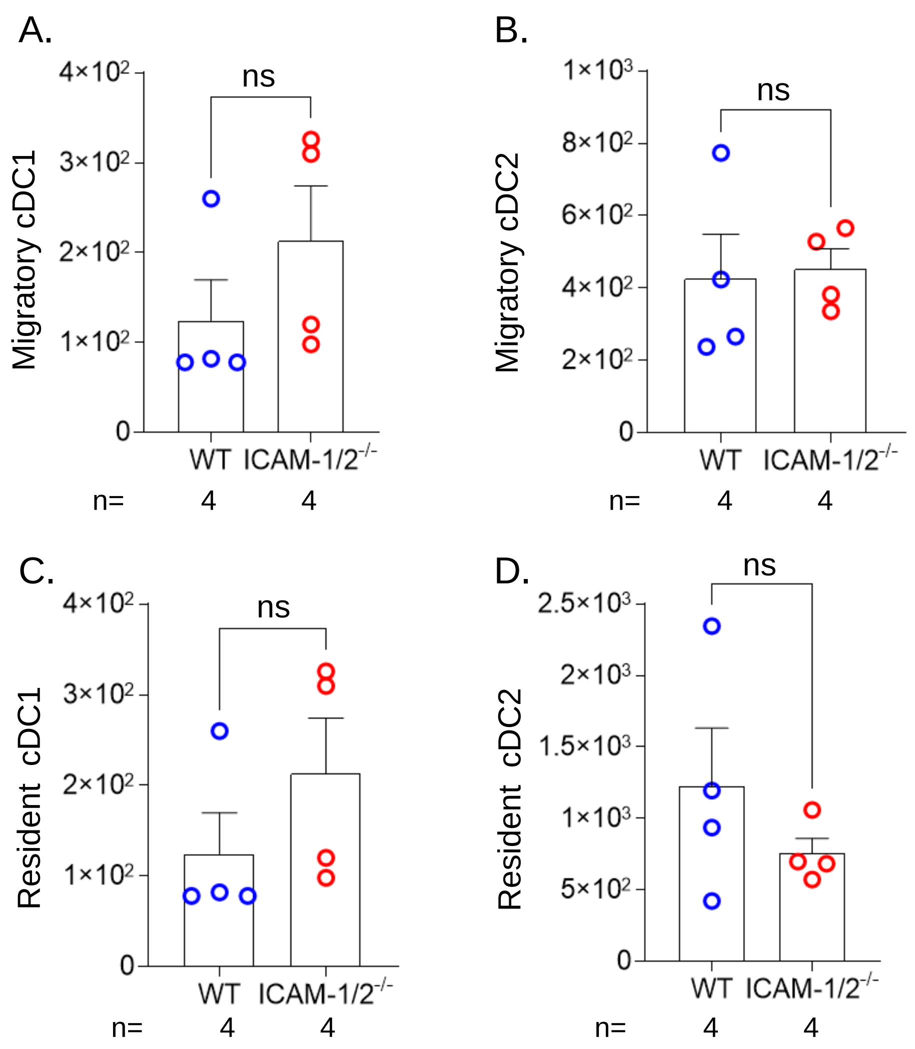

Supplementary Figure 7

Accumulation of distinct cDCs in resting MedLNs of WT and ICAM1/2$^{-/-}$ mice. (A, B) The numbers of migratory (MHCII^{hi}CD11c^{int}) cDC1 (A, CD103^{+}CD11b^{-}) and cDC2 (B, CD103^{-}CD11b^{+}) cells. (C, D) The numbers of resident (MHCII^{int}CD11c^{high}) cDC1 (C, CD8^{+}CD103^{+}CD11b^{-}) and cDC2 (D, CD8^{-}CD103^{-}CD11b^{+}) cells in resting WT (blue) and ICAM-1/2$^{-/-}$ (red) mice. Statistical significance was determined by two-tailed, unpaired Student's t tests. ns, not significant. The numbers of each experimental group are indicated in the graphs. The error bars indicate the SEM of each measurement.

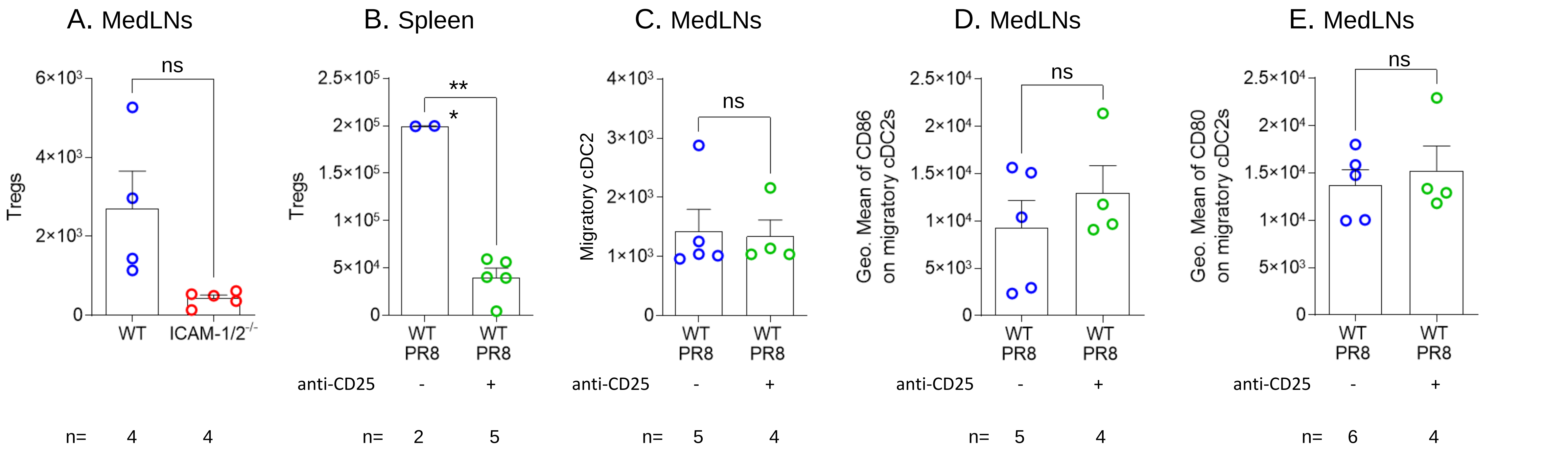

Supplementary Figure 8

Depletion of endogenous Tregs from WT mice with anti-CD25 mAb does not increase cDC2 accumulation in the MedLNs following infection.

(A) The number of Tregs recovered from resting MedLNs of either WT (blue) or ICAM1/2$^{-/-}$ (red) mice. (B) The spleen Treg totals in WT mice, treated once with control (blue, -) or anti-CD25 mAb (P2 mIgG2a (801362.1)) (green, +), that were recovered 3 days post PR8 infection. Mice were administered I.V. with 30ug of the anti-CD25 or control mAbs. (C) The number of cDC2 recovered in the MedLNs of PR8 infected WT mice treated as in (B) are indicated. The surface levels of CD86 (D) and CD80 (E) on the recovered cDC2 of WT mice treated as in (B) were expressed in MFI units. Statistical significance was determined by two-tailed, unpaired Student's t tests. *P < 0.05, ***P < 0.001, ns, not significant. The numbers of each experimental group are indicated in the graphs. The error bars indicate the SEM of each measurement.

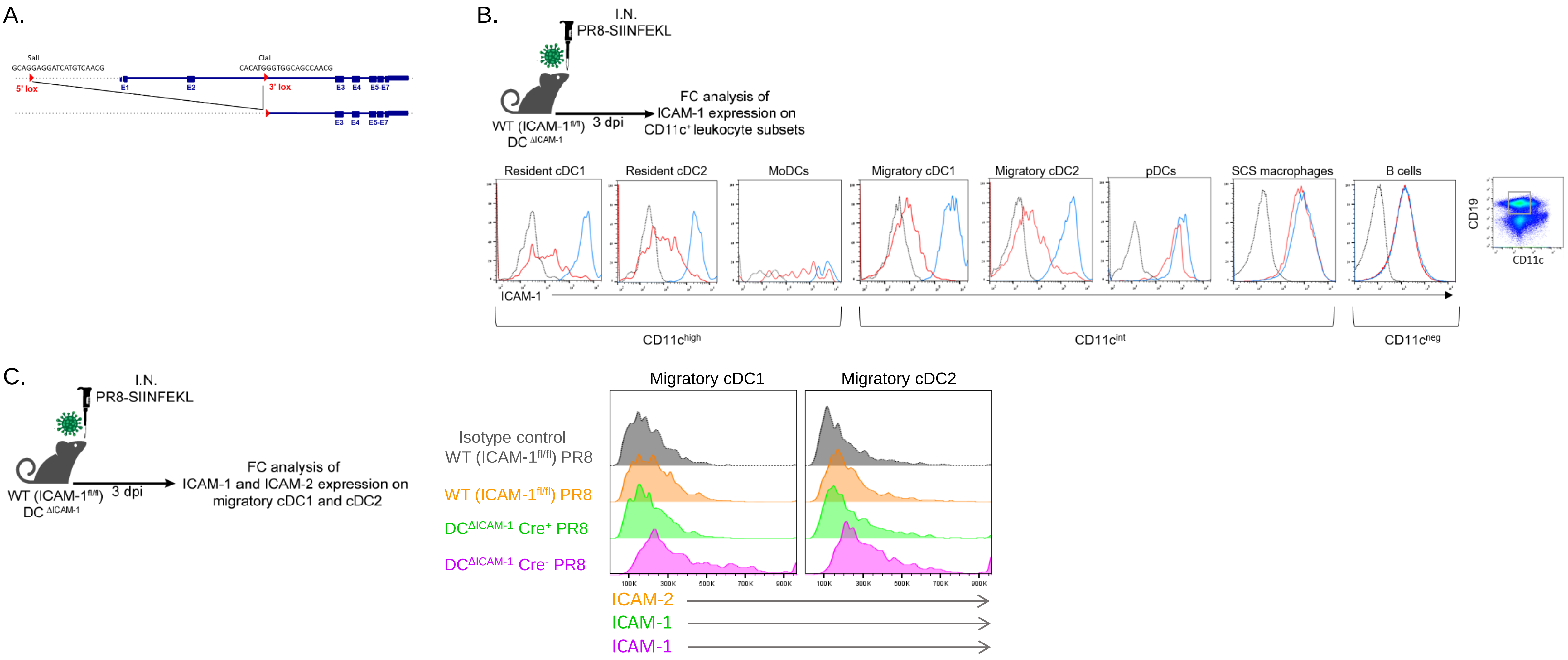

Supplementary Figure 9

ICAM-1 expression on CD11c$^+$ cells is effectively reduced in the MedLN of CD11C-Cre:ICAM-1$^{fl/fl}$ mice. (A) Design of the conditional ICAM-1 locus. Lox sites (red triangles) were inserted using CRISPR-Cas9 upstream of the first exon and downstream of the second exon of ICAM-1. Guide sequences and the restriction site introduced with the Lox screening purposes are shown above each lox site. (B) Timeline of the experimental protocol of infection. A sublethal dose of PR8 influenza was instilled I.N. and 3 days post infection mice were sacrificed, MedLNs were isolated and the surface expression of ICAM-1 on the indicated leukocyte types was determined by flow cytometry. Representation of B cell gating (CD19+CD11c-) (right inset). (C) Timeline of the experimental protocol of infection. A sublethal dose of PR8 influenza was instilled I.N. and 3 days post infection mice were sacrificed, migratory (MHCII^{hi}CD11c^{int}) cDC1 (CD103$^+$CD11b$^-$) and cDC2 (CD103$^-$CD11b$^+$) were isolated (see materials and methods) and the surface expression of ICAM-1 and ICAM-2 on the two subsets was determined by flow cytometry.

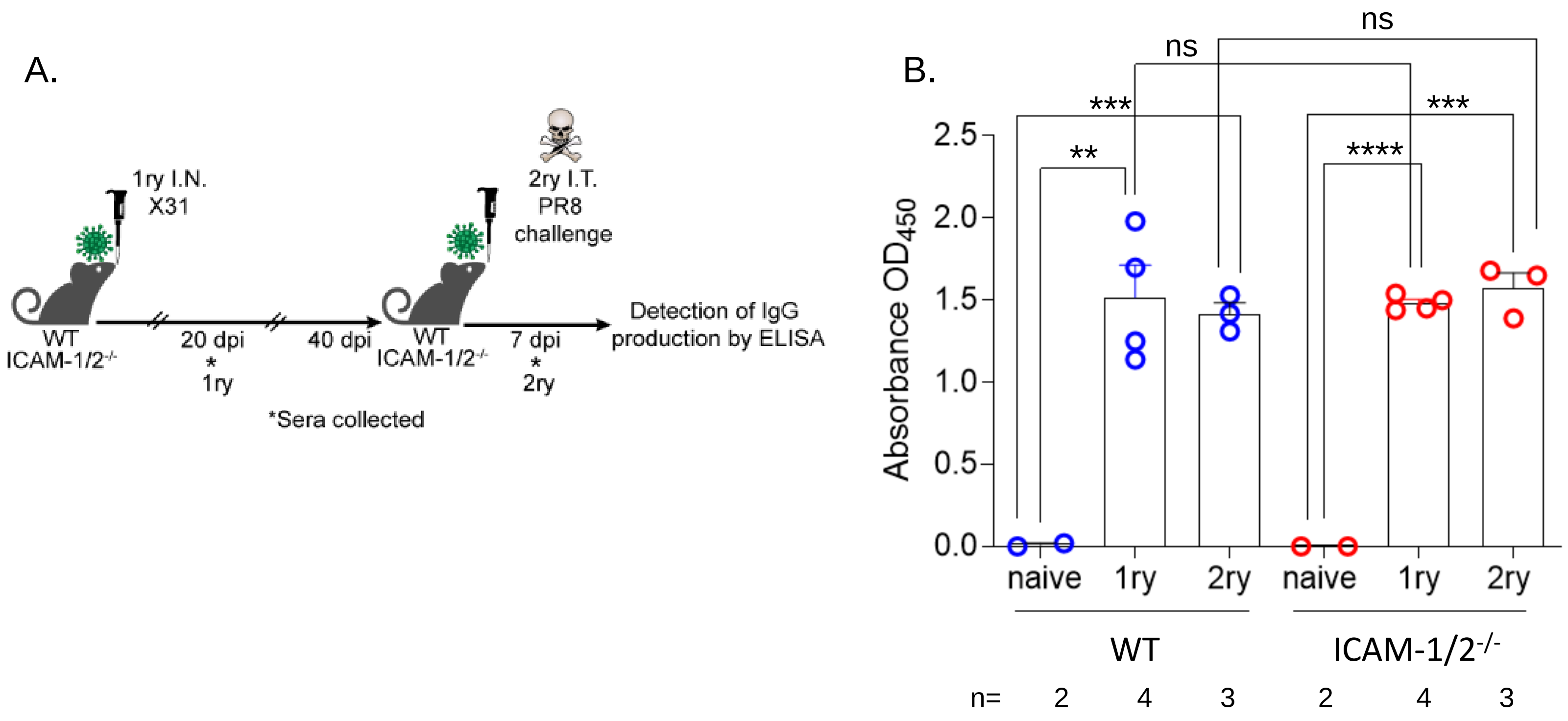

Supplementary Figure 10
Infected ICAM1/2$^{-/-}$ mice produce normal IgG antibodies specific for PR8 influenza. (A) Timeline of the experimental protocol. Mice were infected I.N. with 30 PFU of PR8 influenza. Sera were collected on day 20 post infection and mice were challenged on day 40 with a lethal dose ($3x10^3$ PFU) of PR8 influenza. Sera were collected on day 47 (7 days following the secondary infection). Asterisks indicate sera collection. The levels of PR8 specific IgG antibodies in the sera samples were determined by ELISA. (B) IgG levels detected in the sera of WT and ICAM1/2$^{-/-}$ mice 20 days after the primary PR8 infection (1ry) or 7 days after the secondary PR8 challenge (2ry). Statistical significance was determined by two-tailed, unpaired Student's t tests. **P < 0.01, ***P < 0.001, ****P < 0.0001, ns, not significant. The numbers of each experimental group are indicated in the graph. The error bars indicate the SEM of each measurement.

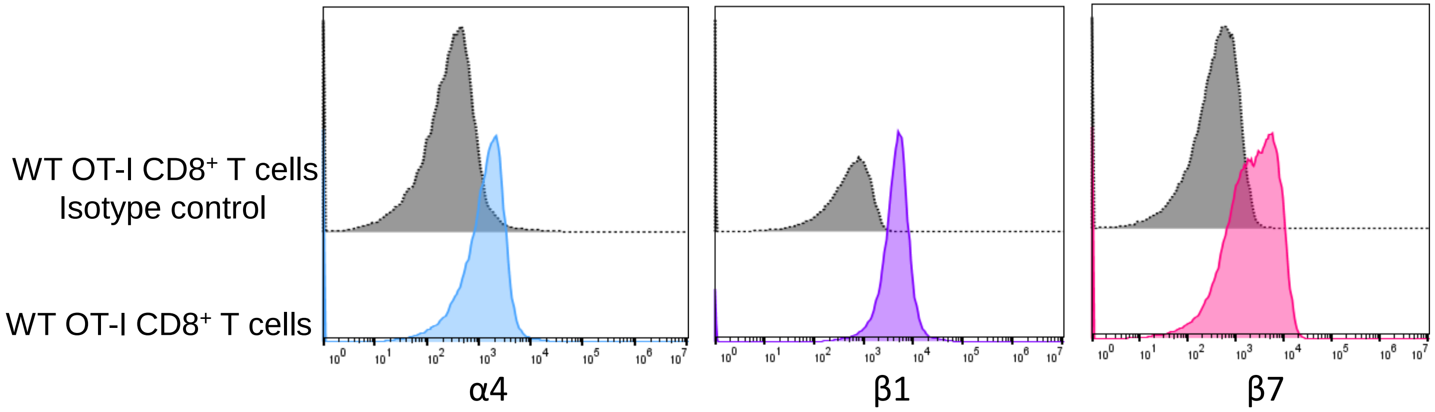

Supplementary Figure 11

OT-I CD8⁺ T cells express comparable levels of the α4 integrins VLA-4 (α4β1) and α4β7. Spleen derived T cells were stained with directly labeled with PE-conjugated anti-α4 integrin (Biolegend, Cat. 103706, clone 9C10 (MFR4.B)), anti-β1 integrin (Biolegend, Cat. 102208, clone HMβ1-1) and anti-β7 integrin (Biolegend, Cat. 321204 , clone FIB504) antibodies.
